# FOXM1 regulates leukemia stem cell quiescence and survival in MLL-rearranged AML

**DOI:** 10.1038/s41467-020-14590-9

**Published:** 2020-02-17

**Authors:** Yue Sheng, Chunjie Yu, Yin Liu, Chao Hu, Rui Ma, Xinyan Lu, Peng Ji, Jianjun Chen, Benjamin Mizukawa, Yong Huang, Jonathan D. Licht, Zhijian Qian

**Affiliations:** 1 Division of Hematology/Oncology, UF Health Cancer Center, University of Florida, Gainesville, FL USA; 2 Department of Medicine, University of Illinois at Chicago, Chicago, IL USA; 3 Institute for Tuberculosis Research, University of Illinois at Chicago, Chicago, IL USA; 4 Department of Pathology, Feinberg School of Medicine, Northwestern University, IL USA; 5 Department of System Biology, City of Hope, CA USA; 6 Cancer & Blood Diseases Institute, Cincinnati Children’s Hospital Medical Center, Cincinnati, OH USA; 7 Department of Medicine, University of Virginia, Charlottestville, VA USA

**Keywords:** Cancer stem cells, Acute myeloid leukaemia

## Abstract

*FOXM1*, a known transcription factor, promotes cell proliferation in a variety of cancer cells. Here we show that *Foxm1* is required for survival, quiescence and self-renewal of MLL-AF9 (MA9)-transformed leukemia stem cells (LSCs) in vivo. Mechanistically, *Foxm1* upregulation activates the Wnt/β-catenin signaling pathways by directly binding to β-catenin and stabilizing β-catenin protein through inhibiting its degradation, thereby preserving LSC quiescence, and promoting LSC self-renewal in MLL-rearranged AML. More importantly, inhibition of FOXM1 markedly suppresses leukemogenic potential and induces apoptosis of primary LSCs from MLL-rearranged AML patients in vitro and in vivo in xenograft mice. Thus, our study shows a critical role and mechanisms of Foxm1 in MA9-LSCs, and indicates that FOXM1 is a potential therapeutic target for selectively eliminating LSCs in MLL-rearranged AML.

## Abstract (web summary)

*FOXM1* is a known transcription factor which promotes cell proliferation in cancer cells. Here, the authors show that *FOXM1* is required for the maintenance of quiescence and self-renewal of leukemia stem cells in MLL-AF9-rearranged acute myeloid leukemia patient and mouse models.

## Introduction

Acute myeloid leukemia (AML) is a fatal form of hematologic malignancies as a consequence of gene mutations and genomic rearrangements that occur in hematopoietic stem/progenitor cells^1^. The standard treatment of most types of AML has not changed since the 1970s, and the low cure rates in AML have not improved. Relapse after initial achievement of complete remission remains a major challenge in AML treatment^1^. Dick and his colleagues showed that AML is organized in a hierarchical fashion, being sustained by leukemia stem cell (LSCs) in vivo^2,3^. Accumulating evidence suggests that LSCs play a key role in the development and maintenance of AML^4,5^. Most LSCs are in a quiescent state, and exhibit a low rate of energy production^6^. This relatively dormant condition likely protects LSCs under restrictive conditions. The LSCs often evade cytotoxic effects of anticancer drugs, which target rapidly proliferating cells^7^. Development of therapeutic strategies for targeting LSCs holds the promise to identify a more effective therapeutic approach to cure AML. Currently, various clinical trials are developing LSC-directed therapies by targeting cell cycle, metabolic, and epigenetic pathways associated with LSCs^8–11^.

FOXM1, which belongs to a large FOX family with more than 55 members, has a conserved DNA binding domain called Winged Helix/Forkhead^12,13^. It was first identified as a key regulator in G1 to S phase transition^14–16^. FOXM1 controls G2 to M phase transition and mitotic progression^17–21^. In addition, *Foxm1* was shown to regulate embryogenesis, organ injury regeneration, and carcinogenesis^22,23^.

FOXM1 gene is overexpressed in a variety of solid tumors^22^. FOXM1 overexpression is often associated with an increased proliferation of tumor cells in lung, colon, prostate, and liver^22^. More recently, FOXM1 was shown to play a critical role in the maintenance of Glioblastoma stem cell^24^. FOXM1 upregulation was also observed in blood cancers including ALL^25^ and myeloma^26^. Inhibition of FOXM1 reduced proliferation in AML leukemia cell lines^27^. In addition, FOXM1 was reported to contribute to chemoresistance in AML, although the molecular mechanisms have not been determined^28,29^. These studies point to the importance of further understanding the role and underlying molecular mechanisms of FOXM1 in LSCs in AML. Mixed lineage leukemia-rearranged (MLL-r) AMLs occur in up to 70% of infant leukemia, and in about 10% of AML^30–32^, and are usually associated with a poor clinical outcome^33^. However, the specific role of FOXM1 in the pathogenesis of MLL-r AML is unknown.

Here we show that high FOXM1 expression is associated with MLL-r AMLs, and that it is required for the maintenance of MLL-r LSCs in human and mouse in vitro and in vivo. Our data reveal that survival of LSCs but not normal HSCs is sensitive to FOXM1 inhibition in both mouse and human. By using both mouse model and patient-derived xenograft (PDX) model, we provide a proof of concept that targeting Foxm1 is a potential LSC-directed treatment for MLL-r AML.

## Results

### FOXM1 upregulation is associated with MLL-r AMLs

**Fig. 1:**
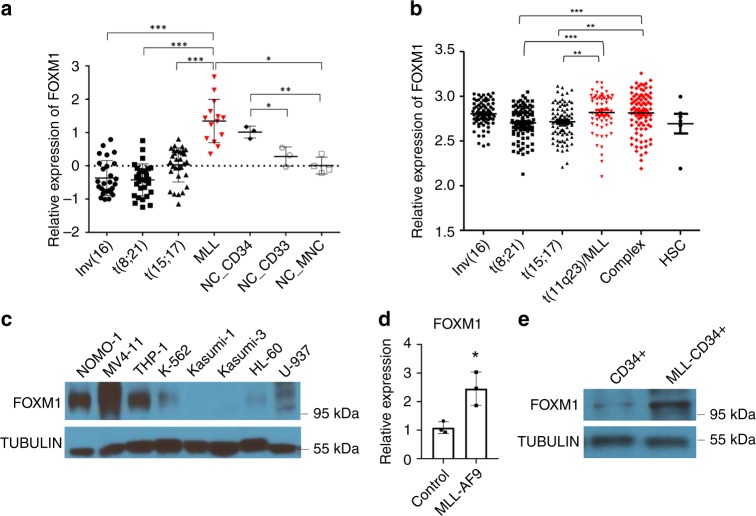
FOXM1 is upregulated in MLL-r leukemia cells. **a**, **b** Comparison of FOXM1 expression among human primary AML cases with MLL rearrangements t(11q23) (MLL) and those without MLL rearrangements (non-MLL) AML cases. t(8;21), t(15;17), and inv(16) are AML subtypes. MLL leukemia includes MLL-AF4 and MLL-AF9. CD34^+^ HSPCs, CD33^+^ myeloid progenitors, and mononuclear cells (MNC) from healthy donors were used as controls. The expression values were log2-transformed and mean centered. The expression data (**a**) and (**b**) were described, respectively, in previous study^34^, and in other datasets of AML patients^35^. **c** Western Blot analysis of FOXM1 expression in human myeloid leukemia cells with different fusion genes. NOMO-1, MV4-11 and THP-1 harbored the MLL rearrangements translocation, which had relatively higher FOXM1 protein level compared to other non-MLL rearrangements cells. **d**, **e** FOXM1 expression in human CD34^+^ cells, which were isolated from cord blood, infected with control plasmid or MLL-AF9-YFP, as determined by quantitative(q)RT-PCR (**d**) or Western Blot analysis (**e**). The average expression level of FOXM1 in the CD34^+^ cells with control plasmid was set as 1 for qRT-PCR. TUBULIN served as the inner control for WB assay. *p < 0.05, **p < 0.01, ***p < 0.001, mean ± s.d., *t*-test. Source data are provided as a Source Data file.

*FOXM1* upregulation was observed in AML patients^27^. However, by analyzing the published microarray dataset^34^, we found that high *FOXM1* expression was associated with MLL-r AML but not AMLs with other common cytogenetic abnormalities including t(8;21), t(5;17) or inv(16) (Fig. 1a). Consistent with this finding, analysis of *FOXM1* expression in other datasets of AML patients^35^ revealed that *FOXM1* expression is significantly increased in MLL-r AML and AMLs with a complex karyotype (Fig. 1b) as compared to AML with other cytogenetic abnormalities. Of note, MV4-11, THP-1, and NOMO-1 leukemia cell lines with presence of *MLL-*fusion genes exhibited high FOXM1 expression compared to leukemia cell lines such as K-562, and Kasumi-3 which lack *MLL*-fusion genes (Fig. 1c). As determined by qRT-PCR and immunoblot, *MA9* significantly induced *FOXM1* expression in human CD34^+^ progenitor cells (Fig. 1d, e).

### *Foxm1* loss significantly delayed MA9-induced AML in vivo

**Fig. 2:**
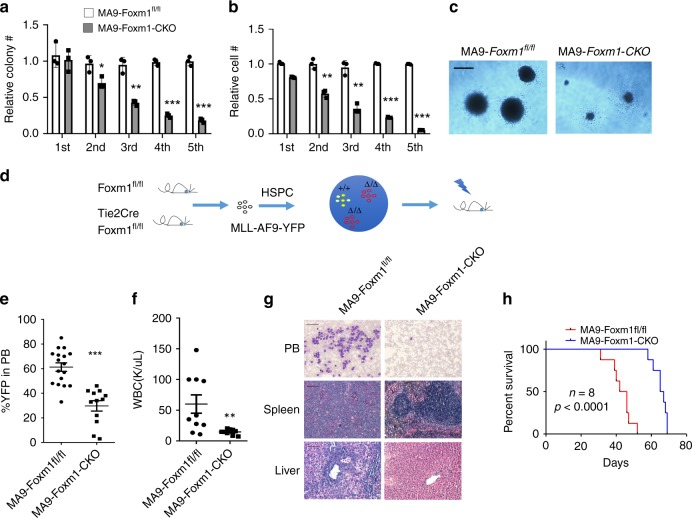
Foxm1 loss delays the development of AML induced by MA9 in vivo. **a** The relative number of colony-forming units. **b** The relative number of cells. **c** Representative images of the colonies for the 5^th^ round of plating are displayed. The MA9 infected Foxm1 CKO or control (Foxm1^fl/fl^) Lin^−^BM cells were resuspended and replated weekly in methocult^TM^ medium containing cytokines, bar = 100 μM. **d** Schematic diagram for generation of MA9-induced Foxm1^fl/fl^ and Foxm1^fl/fl^-CKO AML mouse models. **e** Flow cytometric analysis of engraftment of YFP^+^ cells from MA9-Foxm1^fl/fl^ or MA9-Foxm1-CKO mice 1-month post-transplantation, *n* = 17 MA9-Foxm1^fl/fl^ mice or *n* = 12 MA9-Foxm1-CKO mice. **f** Complete blood count analysis of white blood cells (WBCs) in MA9-Foxm1^fl/fl^ or MA9-Foxm1-CKO mice 1-month post-transplantation, *n* = 10 mice for each group. **g** Wright-Giemsa-stained PB and H&E-stained spleen and liver of the MA9-Foxm1^fl/fl^ or MA9-Foxm1-CKO mice are shown. Bar = 100 μM for PB, bar = 200 μM for spleen and liver. **h** Kaplan–Meier survival analysis of MA9-Foxm1^fl/fl^ or MA9-Foxm1-CKO leukemic mice, *n* = 8 mice for each group. *p < 0.05, **p < 0.01, ***p < 0.001, mean ± s.d., *t*-test or Log-rank (Mantel-Cox) Test for survival curve. Source data are provided as a Source Data file.

qPCR revealed that MA9-induced *Foxm1* expression in mouse myeloid progenitor cells (Supplementary Fig. 1a). We next performed serial replating assay of MA9-transformed stem/progenitor cells with or without *Foxm1*. Conditional *Foxm1* knockout mice (Foxm1^fl/fl^) mice^16^ were crossed with Tie2-Cre mice^36^, to generate Tie2-CreFoxm1^fl/fl^ mice, with conditional deletion of *Foxm1* in HSCs. Bone marrow (BM) cells isolated from Tie2-CreFoxm1^fl/fl^ and control Foxm^fl/fl^ mice were infected with retrovirus-expressing MA9, followed by being plated in MethoCult^TM^ medium. We showed that Foxm1 deletion was incomplete in MA9-Tie2-CreFoxm1^fl/fl^ HSPCs (Supplementary Fig. 1b). Thus, MA9-transduced cells from colonies with validation of *Foxm1* deletion (refer to Foxm1-CKO cells) or *Foxm1*^fl/fl^ colonies were collected (Supplementary Fig. 1c), and the cells were resuspended and replated weekly for five times in MethoCult^TM^ medium. Both the colony-forming units (CFUs) and total cell number derived from MA9-transformed *Foxm1* CKO progenitor cells were decreased gradually as compared to MA9-transduced control wildtype cells after each replating (Fig. 2a, b). At the fifth round of replating, the MA9-transduced Foxm1-CKO progenitor cells gave rise to 80% fewer colonies with smaller size and 90% fewer cells than control cells (Fig. 2a–c), indicating that *Foxm1* KO significantly reduces the self-renewal capacity of MA9-transformed HSPCs in vitro.

We next determined the role of *Foxm1* loss in MA9-induced leukemia in vivo in mice. The same number of MA9-Foxm1-CKO cells or MA9-Foxm1^fl/fl^ cells, collected from the first plating, was transplanted into sublethally irradiated recipient wildtype mice (Fig. 2d) to generate MA9-Foxm1-CKO and MA9-Foxm1^fl/fl^ recipient mice. At 1 month post-transplantation, the average percentage of YFP^+^ MA9-Foxm1^fl/fl^ leukemia cells was markedly higher than that of YFP^+^MA9-Foxm1-CKO leukemia cells (Fig. 2e). We found that MA9-Foxm1^fl/fl^ chimeric mice rapidly developed AML characterized by significantly increased white blood counts as compared to MA9-Foxm1-CKO recipient mice (Fig. 2f). The MA9-Foxm1^fl/fl^ recipient mice developed splenomegaly with an infiltration of leukemia cells into spleen and liver, as well as accumulation of myeloid blast cells in peripheral blood (PB), whereas MA9-Foxm1-CKO recipient mice showed normal structure of spleen and liver without detectable myeloid blast cells in PB (Fig. 2g). Notably, all Foxm1^fl/fl^ recipient mice died within 52 days whereas none of the MA9- Foxm1-CKO chimeric mice died at the same period of time (Fig. 2h). These results suggest that deletion of *Foxm1* significantly delayed the initiation of MA9-transformed leukemia in vivo.

To rule out the possibility that reduced engraftment of MA9-Foxm1-CKO cells as compared to MA9-Foxm1^fl/fl^ cells in recipient mice is due to compromised homing ability of MA9-Foxm1-CKO cells. We performed homing assay to determine whether Foxm1 loss affected the homing ability of MA9-induced leukemia cells. MA9-Foxm1^fl/fl^ and MA9-Foxm1-CKO cells were transplanted into lethally irradiated B6 wildtype mice. As shown in Supplementary Fig. 2, comparable percentage of YFP+/leukemia cells were detected in BM cells from both recipient mice transplanted with MA9-Foxm1^fl/fl^ or MA9-Foxm1-CKO cells, suggesting that loss of *Foxm1* does not affect the homing ability of MA9-induced leukemia cells.

To investigate the role of Foxm1 in a none-MLL-r AML, we determined whether leukemogenic function of AML1-ETO is dependent on Foxm1. AML1-ETO fusion gene results from the t(8;21) translocation, which is one of the most common chromosomal abnormalities identified in AML and AML1-ETO9a is a short isoform of AML1-ETO fusion genes^37^. As determined by serial colony-forming assay, Foxm1-CKO BM cells expressing AML1-ETO9a gave rise to increased number of colonies at the first, second, and third plating as compared to control Foxm1^fl/fl^ BM cells expressing AML1-ETO9a (Supplementary Fig. 3), suggesting that Foxm1 plays an oncogenic role in MLL-AF9-induced AML but not AML1-ETO9a-induced AML.

### Foxm1 is critical for the maintenance of MA9-LSCs

**Fig. 3:**
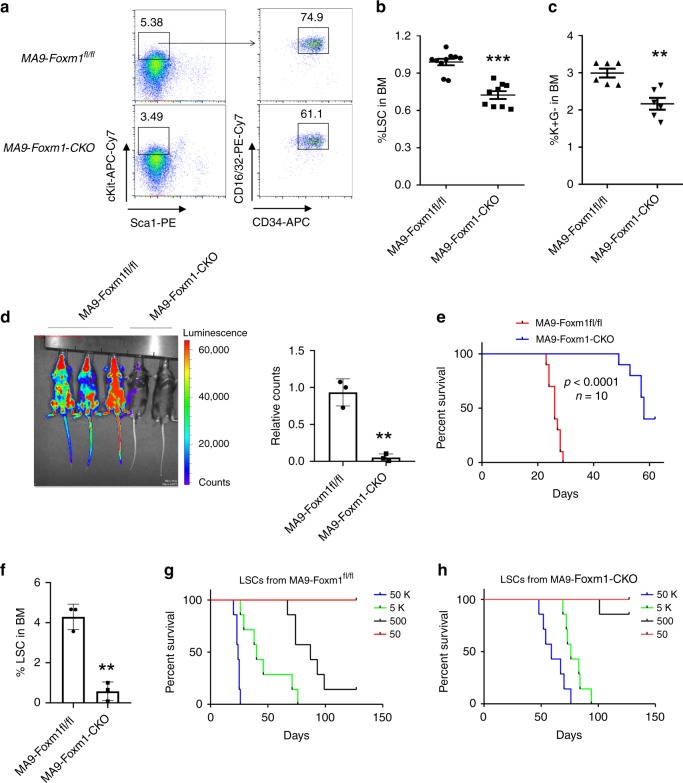
Foxm1 is critical for the maintenance of MA9-transformed LSCs. **a**–**c** Flow cytometric analysis of percentage of LSC (L-GMP: Lin^−^c-Kit^+^Sca1^−^CD34^+^FcR-ϒ^+^) or c-Kit^+^Gr1^−^ in BM cells from MA9-Foxm1^fl/fl^ or MA9-Foxm1-CKO leukemic mice. Gating strategy for L-GMP is shown in (**a**). Mice were sacrificed and BM cells were harvested for analysis when the mice become moribund, *n* = 10 MA9-Foxm1^fl/fl^ mice or *n* = 9 MA9-Foxm1-CKO mice in (**b**), *n* = 6 mice for each group in (**c**) **d** Leukemia burden of secondary recipient mice reconstituted with MA9-Foxm1^fl/fl^ or MA9-Foxm1-CKO BM cells from the primary recipient mice labeled with Luciferase was determined by in vivo imaging system (IVIS). Relative counts are shown. Mice were administrated substrate Luciferin before images were taken, *n* = 3 mice for each group. **e** Kaplan–Meier survival analysis of leukemia maintenance of the secondary recipient mice reconstituted with MA9-Foxm1^fl/fl^ or MA9-Foxm1-CKO BM cells from the primary recipient mice, *n* = 10 mice for each group. **f** Flow cytometric analysis of percentage of LSC in the secondary recipient mice reconstituted with MA9-Foxm1^fl/fl^ or MA9-Foxm1-CKO BM cells from the primary recipient mice, *n* = 3 mice for each group. **g**–**h** Limiting dilution analysis of LSC frequency in the primary MA9-Foxm1^fl/fl^ (**g**) or MA9-Foxm1-CKO (**h**) leukemia mice. 50000, 5000, 500, or 50 L-GMPs sorted from primary MA9-Foxm1^fl/fl^ or MA9-Foxm1-CKO leukemia mice were injected into the recipient mice, *n* = 7 mice for each group. **p < 0.01; ***p < 0.001, mean ± s.d., *t*-test or Log-rank (Mantel–Cox) Test for survival curve. Source data are provided as a Source Data file.

The L-GMPs (Lin^−^c-Kit^+^Sca1^−^CD34^+^FcR-ϒ^+^)^38^ or c-Kit^+^ Gr1^−^BM cells^39^ were defined as LSCs in mouse *MA9* transplant recipient mice. To determine the consequence of *Foxm1* loss on maintenance of MA9-transformed LSCs in vivo, we characterized the MA9-transformed L-GMPs in MA9-Foxm1-CKO or MA9-Foxm1^fl/fl^ recipient mice when the MA9-Foxm1^fl/fl^ recipient mice became moribund. The number of L-GMPs (LSCs) and c-Kit^+^ Gr1^−^ cells were both markedly reduced in MA9-Foxm1-CKO recipient mice compared to control MA9-Foxm1^fl/fl^ recipient mice (Fig. 3a, b, c). To monitor leukemia cells in mice by in vivo imaging, the MA9-Foxm1-CKO or control MA9-Foxm1^fl/fl^ leukemia cells were labeled by luciferase, followed by transplantation into recipient mice. At 1 month post-transplantation, as measured by luciferase bioimaging, leukemia burden in MA9-Foxm1 CKO recipient mice was significantly lower than that in MA9-Foxm1^fl/fl^ recipient mice (Supplementary Fig. 4).

In vivo serial transplantation represents the gold standard for analysis of the long-term function of LSCs^38,40–42^. To evaluate the self-renewal ability of LSCs from primary transplantation mice, we performed secondary transplantation of total BM cells from luciferase-labeled MA9-Foxm1-CKO or control MA9-Foxm1^fl/fl^ primary chimeric mice into the recipient mice receiving a half-lethal dose of irradiation. At 3 weeks post-transplantation, leukemia burden, as determined by in vivo imaging, was reduced dramatically in MA9-Foxm1 CKO recipient mice by more than 10-fold as compared to MA9-Foxm1^fl/fl^ recipient mice (Fig. 3d). Notably, the control MA9-Foxm1^fl/fl^ mice died within 29 days after transplantation while more than 40% of MA9-Foxm1-CKO mice survived beyond 58 days after transplantation (Fig. 3e). As determined by flow cytometric analysis, the frequency of L-GMPs was dramatically decreased in MA9-Foxm1-CKO as compared to MA9-Foxm1^fl/fl^ secondary transplantation mice by 7.5-fold (Fig. 3f) and as compared to an approximately 30% decrease of L-GMPs in primary transplantation recipient mice (Fig. 3a), suggesting that *Foxm1* loss dramatically reduces the self-renewal capacity of LSCs.

To quantitate the functional LSCs in primary MA9-Foxm1-CKO or control MA9-Foxm1^fl/fl^ recipient mice, we performed transplantations with limiting dilutions of sorted YFP^+^ MA9-Foxm1 CKO or control MA9/Foxm1^fl/fl^ L-GMPs from the primary recipient mice. The mice were monitored for survival for 4–5 months post-transplantation. The recipient mice developed AML with a shorter latency when the mice received a higher dose of L-GMPs (Fig. 3g, h). As determined by ELDA software^43^, the frequency of LSCs was reduced by 6-fold in MA9-Foxm1-CKO recipient mice as compared to MA9-Foxm1^fl/fl^ recipient mice. Taken as a whole, our results showed that Foxm1 loss reduced the frequency and self-renewal capacity of MA9-transformed LSCs, indicating that *Foxm1* is essential for the maintenance of MA9-transformed LSCs in mice.

### Foxm1 regulates quiescence and survival of MA9-LSCs

**Fig. 4:**
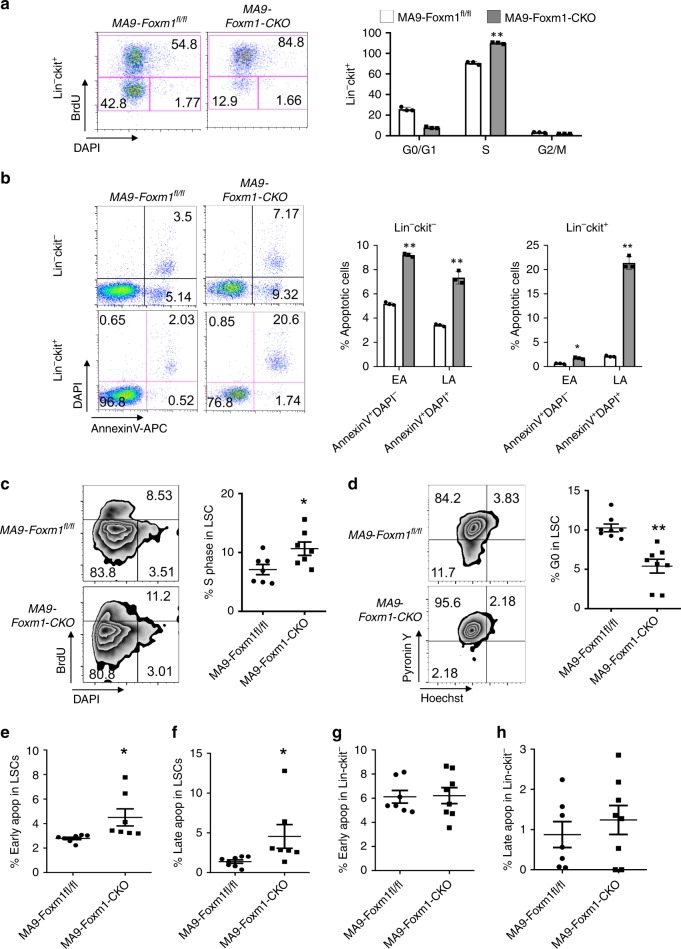
Foxm1 regulates quiescence and survival of MA9-transformed LSCs. **a** Flow cytometric analysis of the cell cycle of LSC-enriched Lin^−^c-kit^+^ population in MA9-Foxm1^fl/fl^ or MA9-Foxm1-CKO cells in vitro. Gating strategy is shown in left panel. Cells were stained with BrdU (determine the S phase) and DAPI (determine the DNA content). **b** Flow cytometric analysis of the apoptosis of LSC-enriched Lin^−^ckit^+^ population and mature myeloid population Lin^−^c-Kit^−^ in MA9-Foxm1^fl/fl^ or MA9-Foxm1-CKO cells in vitro. Gating strategy is shown in left panel. Cells were stained with AnnexinV and DAPI. AnnexinV^+^DAPI^−^ cells represent early apoptotic cells. AnnexinV^+^DAPI^+^ cells represent late apoptotic cells. **c** Flow cytometric analysis of cell cycle of LSC in MA9-Foxm1^fl/fl^ or MA9-Foxm1-CKO primary mice. Gating strategy is shown in left panel. Cells were stained with BrdU (determine the S phase) and DAPI (determine the DNA content), *n* = 7 mice for each group. **d** Flow cytometric analysis of LSC quiescence in MA9-Foxm1^fl/fl^ or MA9-Foxm1-CKO primary mice. Gating strategy is shown in left panel. Cells were stained with DNA Dye Hoechst and RNA Dye PyroninY. Double negative population indicated the G0 phase/quiescence, *n* = 8 mice for each group. **e**–**f** Flow cytometry analyzing the early (**e**) and late (**f**) apoptosis (apop) of LSC in MA9-Foxm1^fl/fl^ or MA9-Foxm1-CKO primary mice, *n* = 8 MA9-Foxm1^fl/fl^ mice or *n* = 7 MA9-Foxm1-CKO mice. **g**–**h** Flow cytometry analyzing the early (**g**) and late (**h**) apoptosis of Lin-cKit- in MA9-Foxm1^fl/fl^ or MA9-Foxm1-CKO primary mice, *n* = 7 MA9-Foxm1^fl/fl^ mice or *n* = 8 MA9-Foxm1-CKO mice, *p < 0.05, **p < 0.01, mean ± s.d., *t*-test. Source data are provided as a Source Data file.

Next, we determined how *Foxm1* regulates cell cycle and survival of MA9-transformed LSCs. The primary Foxm1-CKO or Foxm1^fl/fl^ BM cells were infected with a retrovirus-expressing MA9-MSCV-YFP. We performed cell cycle and survival analysis of the gated YFP^+^Lin^−^c-kit^+^ (LSC-enriched population) from MA9-Foxm1-CKO or MA9-Foxm1^fl/fl^ BM cells. Notably, Lin^−^c-kit^+^ MA9-Foxm1-CKO cells had a significantly increased frequency of cells in S phase as compared to control Lin^−^c-kit^+^ MA9-Foxm1^fl/fl^ cells (Fig. 4a), indicating that MA9-Foxm1-CKO cells are more proliferative than the MA9 control cells. In addition, Foxm1 KO markedly increased the frequency of early (Annexin V^+^DAPI^−^) or late apoptosis in Lin^−^c-kit^+^ (Annexin V^+^DAPI^+^) BM cells by 10-fold while it increased frequency of apoptosis of relatively mature myeloid cell (Lin^−^c-kit^−^) by 2-fold (Fig. 4b), suggesting that LSC-enriched leukemia cells are much more sensitive to absence of *Foxm1* than mature leukemia cells.

We next determined the consequence of Foxm1 loss on the maintenance of quiescence and survival of MA9-transformed LSCs in vivo. As shown in Fig. 4c and Supplementary Fig. 5a, the percentage of LSCs or c-kit^+^Gr1^−^ cells in S phase significantly increased in MA9-Foxm1-CKO as compared to MA9-Foxm1^fl/fl^ recipient mice at 1 month post-transplantation. However, the percentage of mature leukemia cells (lin^−^c-kit^−^, or lin^+^) in S phase was comparable in MA9-Foxm1^−^CKO and MA9-Foxm1^fl/fl^ recipient mice (Supplementary Fig. 5b). The percentage of LSCs or c-kit^+^Gr1^−^ cells in G_0_ phase was significantly decreased by approximately 2-fold in MA9-Foxm1-CKO mice as compared to MA9-Foxm1^fl/fl^ recipient mice (Fig. 4d and Supplementary Fig. 5c). In addition, both the frequencies of early and late apoptotic cells of LSCs but not mature leukemia cells (Lin^−^c-kit^−^) are markedly higher in MA9-Foxm1- CKO recipient mice than those were in MA9-Foxm1^fl/fl^ mice (Fig. 4e–h and Supplementary Fig. 5d). Collectively, *Foxm1* loss reduces the quiescence, and increases proliferation and apoptosis of MA9-transformed LSCs, thereby leading to disruption of maintenance of MA9-transformed LSCs in vivo.

### Foxm1 loss sensitizes LSCs to therapeutic drugs

**Fig. 5:**
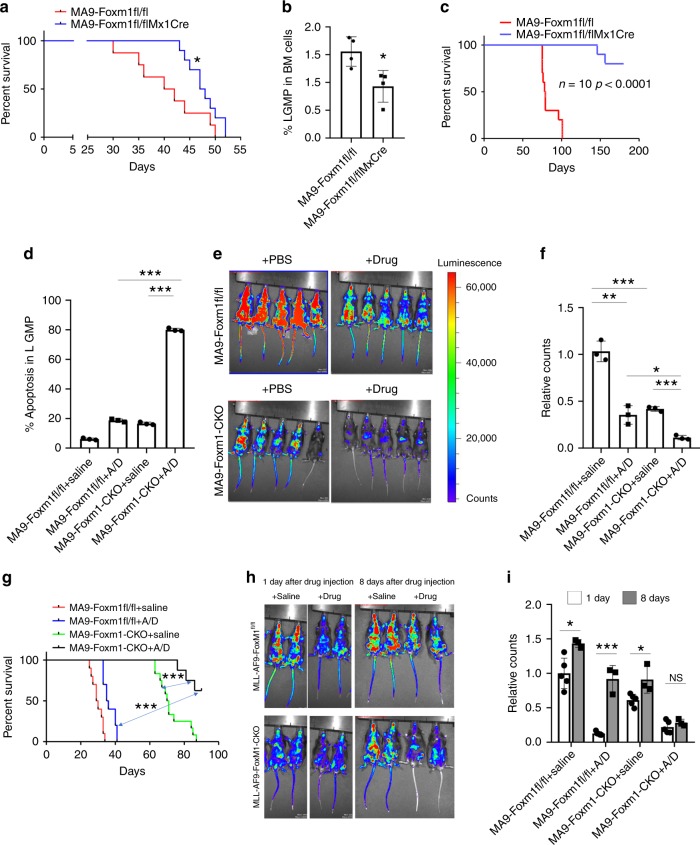
Foxm1 loss increases the sensitivity of LSCs to chemotherapeutic drugs. **a** Kaplan–Meier survival analysis of the primary recipient mice transplanted with Foxm1^fl/fl^ or Mx1-CreFoxm1^f/fl^ BM cells infected with MLL-AF9. Foxm1 deletion was induced by three doses of PIPC injection given every other days after transplantation, *n* = 8 MA9-Foxm1^fl/fl^ mice or *n* = 10 MA9-Foxm1-CKO mice. **b** Flow cytometric analysis of percentage of LSCs in the primary recipient mice transplanted with Foxm1^fl/fl^ or Mx1-CreFoxm1^f/fl^ BM cells infected with MLL-AF9, *n* = 4 mice for each group. **c** Kaplan–Meier survival analysis of the secondary recipient mice reconstituted with MLL-AF9-infected Foxm1^fl/fl^ or Mx1-CreFoxm1^f/fl^ BM cells, which were collected from colonies in methylcellulose medium. Genotype for each colony was confirmed by PCR, *n* = 10 mice for each group. **d** Flow cytometric analysis of apoptosis rate in MA9-Foxm1^fl/fl^ or MA9-Foxm1-CKO LSCs from primary recipient mice treated with saline or 0.5 μM cytarabine (Ara-C) and 15 nM doxorubicin (Doxo). **e**–**f** Leukemia burden of the recipient mice, which were reconstituted with luciferase-labeled MA9-Foxm1^fl/fl^ or MA9-Foxm1-CKO BM cells, and treated with saline or “5 + 3” regimen, was determined by in vivo image system (IVIS) (**e**). Relative counts are shown in (**f**). “5 + 3” regimen: 100 mg/kg Ara-C was administered for consecutive 5 days and for the last 3 days, 3 mg/kg doxorubicin was administered. Mice were injected with substrate luciferin before images were taken, *n* = 3 mice for each group. **g** Kaplan–Meier survival analysis of the recipient mice reconstituted with MA9-Foxm1^fl/fl^ or MA9-Foxm1-CKO BM cells treated with PBS or “5 + 3” regimen, Ara-C and DOXO (A/D), *n* = 10 mice for MA9-Foxm1^fl/fl^ + Saline and A/D group, *n* = 12 mice for MA9-Foxm1-CKO + Saline group or 8 mice for MA9-Foxm1-CKO + A/D group. **h**–**i** Leukemia burden of the recipient mice was determined by in vivo image system (IVIS) at 1 day or 8 days after treatment (**h**). Relative counts of leukemia burden in mice are shown (**i**). The recipients were reconstituted with luciferase-labeled MA9-Foxm1^fl/fl^ or MA9-Foxm1-CKO BM cells, and treated with saline or “5 + 3” regimen, *n* = 5 mice for 1 day or *n* = 3 mice for 8 days group, *p < 0.05, **p < 0.01, ***p < 0.001, mean ± s.d., *t*-test or Log-rank (Mantel–Cox) test for survival curve. Source data are provided as a Source Data file.

We next evaluated the effects of *Foxm1* loss on established MA9-transformed leukemia in vivo. The BM cells isolated from Mx1-CreFoxm^fl/fl^ and control Foxm^fl/fl^ mice were infected with MA9-expressing retrovirus, and transplanted into lethally irradiated recipient mice. When circulating YFP^+^ cells reached 10% in PB, excision of *Foxm1* in the recipient mice were induced by three doses of poly(I:C) injection. The MA9-Mx1-CreFoxm1^Δ/Δ^ recipient mice had a prolonged survival with a reduced percentage of LSCs as compared to MA9-Foxm^fl/fl^ recipient mice (Fig. 5a, b). BM cells isolated from MA9-Mx1-CreFoxm1^Δ/Δ^ recipient mice gave rise to a significantly lower number of CFUs than the BM cells did from control MA9-Foxm1^fl/fl^ mice (Supplementary Fig. 6a). However, significant number of MA9-Mx1-creFoxm1^fl/fl^ escaped from excision of *Foxm1* (Supplementary Fig. 6b) and likely underwent clonal selection to promote progression of AML in the recipient mice. Thus, the cells from the colonies with excision of Foxm1 were collected and transplanted into recipient mice to generate secondary transplantation mice. Notably, the MA9-Foxm1^fl/fl^ recipient mice all died within 100 days after transplantation, while the majority of MA9-Mx1-CreFoxm1^Δ/Δ^ recipient mice survived beyond 200 days (Fig. 5c), suggesting that deletion of Foxm1 eliminated the established MA9-transformed LSCs in vivo.

Emerging evidence suggests that LSCs play a critical role in drug resistance in AML^5^. We next determined whether deletion of *Foxm1* increases the sensitivity of MA9-transformed LSCs to treatment of chemotherapeutic drugs. We found that combination treatment of MA9-Foxm1^fl/fl^ LSCs with DOXO and Ara-C induced a 3-fold increase in apoptosis compared to untreated Foxm1^fl/fl^ LSCs (~5% to ~15%). MA9-Foxm1-CKO LSCs had a higher basal apoptosis rate without treatment than Foxm1^fl/fl^ LSC of ~15% that increased 6-fold to nearly 90% after drug treatment (Fig. 5d). The results indicate that *Foxm1* loss and chemotherapeutic drugs have a synergistic effect on induction of cell apoptosis in MA9-transformed LSCs. However, we did not detect a synergistic effect on induction of cell apoptosis of MA9-Foxm1-CKO leukemia cells (Supplementary Fig. 6c). Next, we transplanted the luciferase-labeled MA9-Foxm1-CKO and MA9-Foxm1^fl/fl^ leukemia cells into recipient mice receiving a half lethal dose of irradiation. The recipient mice received treatment of “5 + 3” (i.e. DOXO and Ara-C) regimen or Saline. Chemotherapy treatment of MA9-*Foxm*^fl/fl^ decreased leukemia burden and reduced the percentage of circulating leukemia cells by about 60% (Fig. 5e, f and Supplementary Fig. 6d). MA9-Foxm1-CKO recipient mice had less leukemia burden in animals and the chemotherapeutic effect was enhanced with circulating cells decreased from approximately 40% in untreated animals to 10% in treated animals. Consistent with the observation that MLL-r AML patients are resistant to chemotherapeutic treatment^44^, the recipient mice received BM cells from primary MA9-Foxm1^fl/fl^ mice poorly responded to treatment of DOXO and Ara-C (Fig. 5g). Flow cytometric analysis revealed that total leukemia cells (Supplementary Fig. 6e) in BM were markedly decreased whereas LSC frequency (Supplementary Fig. 6f) was increased and total LSCs (Supplementary Fig. 6g) were comparable in MA9-Foxm1^fl/fl^ mice with chemotherapeutic drug treatment as compared to the mice without drug treatment. These results indicate that LSCs are more resistant to chemotherapeutic drugs in MA9 mice. Interestingly, we found that *Foxm1* loss together with treatment of DOXO and Ara-C significantly prolonged survival of MA9-driven AML in vivo (Fig. 5g). To determine whether *Foxm1* deletion delays relapse after chemotherapy in MA9 leukemia mice, we treated cohorts of MA9-Foxm1-CKO and MA9-Foxm1^fl/fl^ recipient mice with DOXO and Ara-C. As shown in Fig. 5h, i, 1 day after chemotherapeutic drug treatment, luciferase-labeled MA9-Foxm1^fl/fl^ and MA9-Foxm1-CKO leukemia cells were decreased significantly as determined by in vivo imaging. However, 8 days after treatment the number of MA9-Foxm1^fl/fl^ leukemia cells were significantly increased in vivo whereas MA9-Foxm1-CKO leukemia cells showed no significant increase. These results showed that loss of Foxm1 increased sensitivity of MA9-LSCs to chemotherapeutic drugs, thereby delaying relapse in MA9 mice.

### Foxm1 regulates multiple molecular pathways in LSCs

**Fig. 6:**
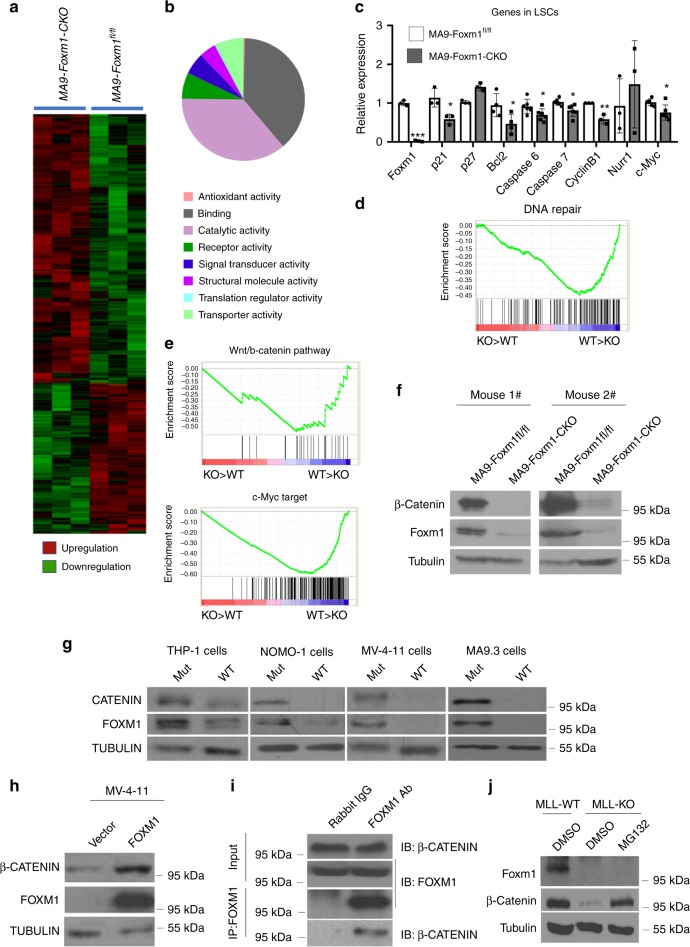
Molecular pathways underlying the role of Foxm1 in LSCs. **a** Heat map of the expression of 546 Foxm1-regulated genes upregulated (red) or downregulated(green) twofold or more (*P* ≤ 0.05) in LSCs from Foxm1-KO MA9-leukemia mice (MA9-Foxm1-KO-LSCs) relative to LSCs from MA9-leukemia mice (MA9-Foxm^fl/fl^ LSCs). **b** Pie charts show the distribution of 546 genes which are differentially expressed in Foxm1-deficinet and control LSCs into functional groups. **c** Quantitative RT-PCR analysis of selected genes in LSCs; results were normalized to those of Actb and are presented relative to those of Foxm1^fl/fl^ control LSCs. **d**–**e** Gene-set-enrichment analysis of selected gene sets (12,000 transcripts) encoding products related to DNA repair pathways (**d**), the Wnt/β-catenin signaling (**e**, top) or that are targeted by c-Myc (**e**, bottom), presented as enrichment score as well as genes “positively correlated” with Foxm1 deficiency (KO > WT) or “negatively correlated” with Foxm1 sufficiency (WT > KO) in MA9-LSCs. **d**, *P* < 0.0001; **e** top, *P* < 0.001; **e** bottom; *P* < 0.001. **f** Western Blot analysis of Foxm1 and β-catenin expression in primary leukemia cells from MA9-foxm1^fl/fl^ (control) and MA9-Foxm1-CKO mice. **g** Western Blot analysis of FOXM1 and β-CATENIN expression in human leukemia cells with expression of MLL fusion genes. The cells were treated with 40 μM FOXM1-specific peptide or mutant peptide. **h** Western Blot analysis of FOXM1 and β-CATENIN expression in MV4-11 human leukemia cells with expression of FOXM1 or control vector. **i** Co-immunoprecipitation assay of endogenous β-CATENIN and FOXM1 in MV4-11 human leukemia cell lines. **j** Western Blot analysis of Foxm1 and β-catenin in BM cells from MA9-Foxm1^fl/fl^ and MA9-Foxm1 CKO leukemia mice. The cells were treated with 20 μM MG132 or DMSO for 6 h, *p < 0.05, **p < 0.01, ***p < 0.001, mean ± s.d., *t*-test. Source data are provided as a Source Data file.

To gain insights into the molecular mechanisms underlying the role of Foxm1 in LSCs, we performed global gene expression analysis of LSCs from three pairs of MA9-transduced Foxm1-CKO and Foxm1^fl/fl^ primary recipient mice by RNA-seq. Five hundred and forty six genes were differentially expressed in LSCs with presence and absence of *Foxm1* (*p* < 0.05) (Fig. 6a). The differentially expressed genes were classified into eight functional groups, including genes encoding products with antioxidant activity, nucleic-binding, catalytic activity, receptor activity, signal transducer activity, structural molecular activity, translation regulator activity, and transporter activity (Fig. 6b), suggesting that a variety of molecular and cellular processes have been altered in Foxm1-CKO LSCs. In comparison of the differentially expressed genes in Foxm1 KO and wildtype HSCs^45^, 10 overlapping genes were commonly downregulated following Foxm1 KO in both HSCs and LSCs (Supplementary Fig. 7a). Consistent with RNA-seq analysis, qPCR revealed that *Ccnb1*(*Cyclin B1*), a known downstream target of *Foxm1*, was downregulated in both Foxm1-CKO LSCs and HSCs. However, expression of Nurr1, which mediates the function of Foxm1 in regulation of HSC quiescence, was unchanged in Foxm1-CKO and control LSCs (Fig. 6c). Thus, these data suggest that distinct downstream pathways are controlled by Foxm1 in LSCs as compared to normal HSCs. p21 is indispensable for maintaining LSC self-renewal while Bcl2 inhibition selectively eradicates LSCs^6,46^. Supporting our observation that MA9-Foxm1-CKO LSCs had a decrease in quiescence as well as an increase of apoptosis compared to MA9-LSCs, p21 and Bcl2 are both significantly downregulated in MA9-LSCs with deletion of Foxm1, as confirmed by qRT-PCR (Fig. 6c). In addition, the other genes including *Caps 6*, *Caps 7* which are involved in mitochondrial-mediated apoptosis showed decreased expression in MA9-Foxm1-CKO LSCs as compared to MA9 LSCs (Fig. 6c). Next, we performed gene-set-enrichment analysis to determine whether an a priori-defined set of genes showed significantly different expression in MA9-Foxm1-CKO LSCs as compared to MA9-LSCs. The result revealed that the set of genes which were expressed at higher level in MA9 LSCs as compared to MA9-Foxm1-CKO LSCs showed enrichment for a gene set associated DNA repair (Fig. 6d), suggesting that increased Foxm1 expression may enhance DNA repair activities in MA9 LSCs. The Wnt/β-catenin signaling pathway plays an essential role in the maintenance of LSCs^39,47^. Interestingly, the set of genes that are associated with the Wnt/β-catenin signaling, or targeted by c-Myc, a downstream regulator of Wnt/β-catenin signaling, were enriched in the upregulated genes in MA9-LSCs (Fig. 6e). Immunoblot and qPCR analysis of primary leukemia cells from both MA9 or MA9-Foxm1-CKO recipient mice revealed that β-catenin protein level but not mRNA levels were dramatically decreased in MA9-Foxm1-CKO BM cells as compared to MA9 BM cells (Fig. 6f and Supplementary Fig. 7b). A peptide form of the p19^ARF^ protein, (D-Arg)_9_ –p19^ARF^ 26-44 was reported to be sufficient to sequester Foxm1 to the nucleolus and effectively inhibit its transcriptional activity^18^. Concordant with these findings, in MA9-transformed leukemia cells in mice, we found that inhibition of FOXM1 by the FOXM1-specific peptide but not FOXM1-mutant peptide significantly reduced β-CATENIN expression in human leukemia cell lines including MV4-11, THP-1, and NOMO-1 cells as well as MA9.3 cells (Fig. 6g and Supplementary Fig. 7c). Furthermore, FOXM1 overexpression significantly increased the expression of β-catenin in MV4-11 cells (Fig. 6h). As shown in Fig. 6i, endogenous FOXM1 interacted with β-CATENIN in MV4-11 cells. Of note, MG132, which is a proteasome inhibitor, restored the β-CATENIN expression in MA9-Foxm1-CKO BM cells from the recipient mice (Fig. 6j). Interestingly, the endogenous polyubiquitination of β-catenin was significantly increased in MA9-Foxm1-CKO BM cells as compared to MA9-Foxm1^fl/fl^ BM cells (Supplementary Fig. 7d). In addition, we found that overexpression of Foxm1 in mice wildtype BM cells did not increase the expression of β-catenin (Supplementary Fig. 7e, f). As determined by flow cytometric analysis (Supplementary Fig. 7g, h), Foxm1 loss did not destabilize β-catenin in normal HSC in vivo. These results showed that in leukemia cells but not in wildtype hematopoietic stem/progenitor cells Foxm1 stabilized β-catenin protein by inhibiting polyubiquitination of β-catenin, thereby preventing its degradation by proteasomes. This indicates a cell-context-dependent role of Foxm1 in regulation of β-catenin stability. To determine whether Foxm1-mediated stabilization of β-catenin is dependent on its binding ability to β-catenin instead of its transcriptional activity, we generated two Foxm1 mutants including Foxm1ΔCat with a deletion of β-catenin binding domain and Foxm1DNAMut containing mutated DNA binding domain according to the published reference^24^. The expression of full length Foxm1 and both foxm1 mutants in MV4-11 cells were confirmed by western blot analysis (Supplementary Fig. 8a). We showed that MV4-11 cells expressing full length Foxm1 or Foxm1 DNAMut but not Foxm1ΔCat had an increased level of β-CATENIN as compared to the MV4-11 cells with control vector (Supplementary Fig. 8a), suggesting that interaction between FOXM1 and β-CATENIN but not its transcriptional activity is critical for Foxm1-mediated stabilization of β-catenin. β-catenin is essential for the maintenance of MA9-induced LSCs^39,47^. To determine whether leukemogenic activity of Foxm1 is dependent on its binding ability to β-catenin, we re-expressed full length *Foxm1* or *Foxm1* mutants in Foxm1-CKO BM cells with expression MA9 fusion gene. As we expected, full length *Foxm1* can fully rescued the reduced colony-forming ability of MA9-Foxm1-CKO cells. Of note, both *Foxm1ΔCat* and *Foxm1DNAMut* increased colony-forming ability of MA9-Foxm1-CKO cells (Supplementary Fig. 8b, c), indicating that the oncogenic function of Foxm1 is partially dependent on the binding ability of Foxm1 to β-catenin and its transcriptional activity. Taken together, our data suggest that increased expression of *Foxm1* likely enhances the self-renewal and survival of MA9 LSCs through multiple molecular pathways including the Wnt/β-catenin pathway, thereby contributing to the maintenance and drug resistance of MA9-induced leukemia.

### MA9 does not directly control Foxm1 expression

To determine whether MLL fusion gene regulates *Foxm1* gene expression by directly binding to *Foxm1* gene promoter region, we have subcloned the 3.3 kb promoter region of *Foxm1* (3.3 kb upstream of transcription start site) into luciferase reporter vector pGL3.0 and performed luciferase reporter assay. As shown in Supplementary Fig. 9a, we showed that overexpression of MA9 did not activate the *Foxm1* gene promoter activity. Consistent with our result from luciferase report assay, analysis of published MLL-fusion gene ChIP-seq databases^48–50^, revealed that there are no potential MLL-fusion gene binding sites present in the *Foxm1* gene promoter region. To evaluate the possibility that upregulation of Foxm1 in MLL-r cells is a consequence of β-catenin activation by MLL-fusion genes, we expressed shRNAs against β-CATENIN in MV4-11 cells. The β-CATENIN-specific shRNAs but not scramble-shRNA efficiently inhibited β-catenin expression in MV4-11 (Supplementary Fig. 9b). However, both β-CATENIN-specific shRNAs barely affect the expression of Foxm1 in the same cells (Supplementary Fig. 9b), suggesting that β-CATENIN does not affect FOXM1 expression in MLL-r leukemia cells.

### Inhibition of FOXM1 suppresses MLL-induced leukemogenesis

**Fig. 7:**
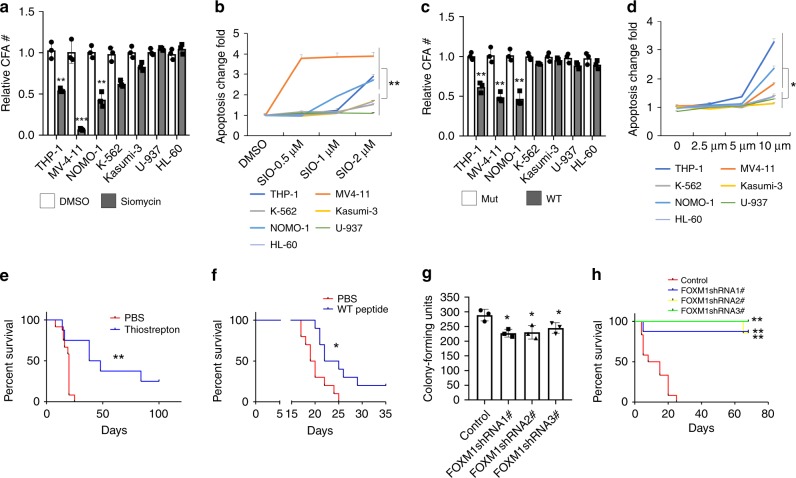
Inhibition of FOXM1 suppressed MLL-r-induced leukemogenesis. **a**, **b** Relative colony-forming units (**a**) and frequency of apoptosis (**b**) of leukemia cells treated with DMSO or 1 μM Siomycin A. MV4-11 and THP-1 and NOMO-1cell lines express MLL fusion genes while K-562, Kasumi-3, HL-60, and U-937 cells do not express MLL-fusion genes. **c**, **d** Relative colony-forming units (**c**) and frequency of apoptosis (**d**) of leukemia cells treated with WT FOXM1-specific ARF26-44 peptide or mutant ARF37–44 peptide. For colony-forming assay, the cells grew in Methocult^TM^ medium containing 2 μM Siomycine, DMSO, 10 μM Foxm1-peptide or mutant peptide for 7 days. The apoptosis frequencies (**b**) were presented relative to those of cells treated with DMSO. The apoptosis frequencies (**d**) were normalized to those of cells treated with mutant peptide at each concentration and presented relative to those of cells treated with mutant peptide. **e** Kaplan–Meier survival analysis of xenografted mice reconstituted with GFP-labeled MV4-11 cells. The mice were treated with 50 mg/kg thiostrepton or vehicle PBS for 7 days, *n* = 12 mice for PBS or *n* = 8 for thiostrepton group. **f** Kaplan–Meier survival analysis of xenografted mice reconstituted with GFP-labeled MV4-11 cells. The mice were treated with 10 mg/kg FOXM1-specific peptide or vehicle PBS for 14 days, *n* = 10 mice for each group. **g** Colony-forming assay for MV4-11 cells infected with inducible PLKO.1-Tet-on FOXM1-shRNA or control vector. 2 μg/ml doxycycline was added in the methylcellulose medium to induce shRNA expression. **h** Kaplan–Meier survival curve of xenografted mice reconstituted with 2 × 10^4^ MV4-11 cells infected with inducible PLKO.1-Tet-on FOXM1-shRNA or control vector. 2 mg/ml doxycycline and 10 mg/ml sucrose were added in drinking water for recipient mice before transplantation, and were maintained throughout their lifetime to induce FOXM1 shRNA expression in vivo, *n* = 12 mice for control group, *n* = 8, 7 or 7 for FOXM1 shRNA 1, 2, or 3 expressing group, respectively, *p < 0.05, **p < 0.01, ***p < 0.001, mean ± s.d., *t*-test or Log-rank (Mantel–Cox) Test for survival curve. Source data are provided as a Source Data file.

To further elucidate the role of FOXM1 in human AML cells, we determined the effects of FOXM1 inhibitors on MV4-11, THP-1, and NOMO-1 leukemia cells with expression of MLL-fusion genes. As shown in Fig. 7a, b, the thiazole antibiotic Siomycin A, a known *FOXM1* inhibitor in human cancer cells^51–54^, significantly inhibited colony-forming ability and induced apoptosis of MV4-11, THP-1, and NOMO-1 but not that of K-562, Kasumi-3, HL-60, and U-937 (Fig. 7a, b). Additionally, the FOXM1-specific ARF26-44 peptide significantly inhibited colony-forming ability and induced apoptosis of MV4-11, NOMO-1, and THP-1 but not control cell lines K-562, Kasumi-3, HL-60, and U-937 as compared to mutant ARF37-44 peptide (Fig. 7c, d). To determine whether FOXM1 inhibition suppresses the MV4-11 leukemia cell grown in vivo, we transplanted GFP-labeled MV4-11 cells into NSGS mice (NOD/SCID/IL2rγ^null^ mice expressing human SCF, GMCSF, and IL3)^55^, to generate cohorts of xenografted mice, followed by treatment with the FOXM1-specific peptide or the natural product thiostrepton, which effectively attenuated FOXM1 expression in human cancer cells in vitro and in vivo in xenograft mice^25,51,54,56^. As determined by flow cytometric analysis, the percentage of GFP-labeled MV4-11 in PB was significantly decreased in xenograft mice with treatment of FOXM1 inhibitors as compared to control xenograft mice (Supplementary Fig. 10a, b). Furthermore, inhibition of FOXM1 by either Thoistrepton or the FOXM1-specific peptide significantly prolonged survival of MV4-11 xenograft mice (Fig. 7e, f). As thoistrepton or FOXM1-specific peptide may have off-target effects, we next knocked down FOXM1 in MV4-11 using three FOXM1-specific shRNAs targeting different sequences of FOXM1. All three FOXM1-specific shRNAs significantly depleted FOXM1 and inhibited cell growth (Supplementary Fig. 10c–e) and colony-forming ability of MV4-11 (Fig. 7g) in vitro. The MV4-11 cells expressing FOXM1-specific shRNAs, or control vector were transplanted into NSG mice and expression of FOXM1-specific shRNA in vivo was induced by doxycycline (DOX). The xenograft mice reconstituted with MV4-11 cells expressing the control vector died within 25 days while all the xenograft mice reconstituted with MV4-11 cells expressing FOXM1 shRNAs survived beyond 70 days after transplantation (Fig. 7h). Together, our results indicated that inhibition of FOXM1 strongly suppressed the growth of MV4-11 cells in vivo.

### Inhibition of FOXM1 suppressed LSCs from MA9 AML patients

**Fig. 8:**
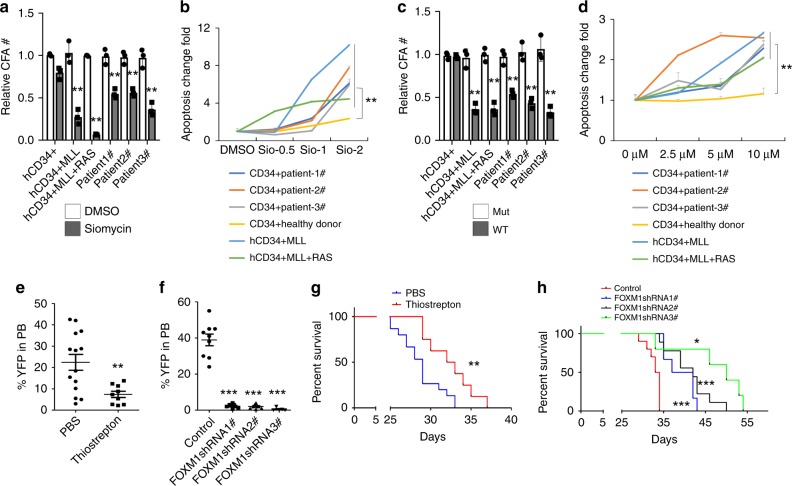
Inhibition of FOXM1 suppressed MA9-transformed CD34^+^ cells. **a, c** Colony-forming assay for hCD34^+^ MLL cells (human CD34^+^ transformed by MLL-AF9), hCD34^+^ MLL + RAS cells (human CD34^+^ transformed by MLL-AF9 and RAS), primary CD34^+^ cells from three patients as well as normal hCD34^+^ cells from healthy donor treated with DMSO or 1uM Siomycin (**a**) or with 10 μM FOXM1 Mut or WT peptide (**c**). **b**, **d** Flow cytometric analysis of apoptosis frequency of hCD34^+^ MLL cells, hCD34^+^ MLL + RAS cells, primary CD34^+^ cells from three patients as well as normal hCD34^+^ cells from healthy donor treated with DMSO or different concentration of Siomycin A(**b**) or with different concentration of FOXM1 WT and Mut peptide (**d**). Apoptosis fold change was determined by comparison of Siomycin with DMSO treatment (**b**) or by comparison of FOXM1 WT peptide with Mut peptide (**d**). **e** Flow cytometric analysis of YFP percentage in PB cells collected from xenografted mice reconstituted with MA9.3 cells (human CD34^+^ transformed by MLL-AF9-YFP). The mice were treated with 50 mg/kg thiostrepton or vehicle PBS for 7 days. **f** Flow cytometric analysis of YFP percentage in PB cells collected from xenografted mice reconstituted with MA9.3 cells infected with inducible PLKO.1-Tet-on FOXM1-shRNA or control vector. 2 mg/ml doxycycline and 10 mg/ml Sucrose were added in drinking water for recipient mice before transplantation, and were maintained throughout their lifetime to induce shRNA expression in vivo. **g** Kaplan–Meier survival curve of xenografted mice reconstituted with MA9.3 cells. The mice were treated with 50 mg/kg thiostrepton or vehicle PBS for 7 days, *n* = 15 mice for PBS or *n* = 8 mice for thiostrepton treatment group. **h** Kaplan–Meier survival curve of xenografted mice reconstituted with MA9.3 cells infected with inducible PLKO.1-Tet-on FOXM1-shRNA or control vector. 2 mg/ml doxycycline and 10 mg/ml Sucrose were added in drinking water for recipient mice before transplantation, and were maintained throughout their lifetime to induce shRNA expression in vivo, *n* = 10 mice for control group or *n* = 6, 9, or 5 mice for FOXM1 shRNA 1, 2, or 3 expressing groups, respectively, *p < 0.05, **p < 0.01, ***p < 0.001, mean ± s.d., *t*-test or Log-rank (Mantel–Cox) Test for survival curve. Source data are provided as a Source Data file.

To determine the effects of FOXM1 inhibition on LSCs enriched population, we treated the MA9.3 cells, MA9.3/NRAS cells, CD34^+^ stem/progenitor cells from AML patients as well as normal CD34^+^ stem/progenitor cells with Siomycin A or FOXM1-specific ARF26-44 peptide or mutant ARF37–44 peptide. MA9.3 cells or MA9.3/NRAS cells were derived from human CD34^+^ cells transformed by MA9 or MA9 and NRAS (G12D), respectively, and produced AML in NOD/SCID mice^57^. Notably, FOXM1 inhibition by either Siomycin A or FOXM1-specific peptide significantly inhibited colony-forming ability and induced apoptosis of MA9.3 cells, MA9.3RAS and primary CD34^+^ cells from MLL-r AML patients but not normal CD34^+^ cells (Fig. 8a–d). In a complementary experiment, the FOXM1 gene was knocked down by three FOXM1-specific shRNAs in MA9.3 cells (Supplementary Fig. 11a). All three FOXM1-specific shRNAs markedly inhibited cell growth, colony-forming ability and induced apoptosis of MA9.3 cells (Supplementary Fig. 11b–d).

**Fig. 9:**
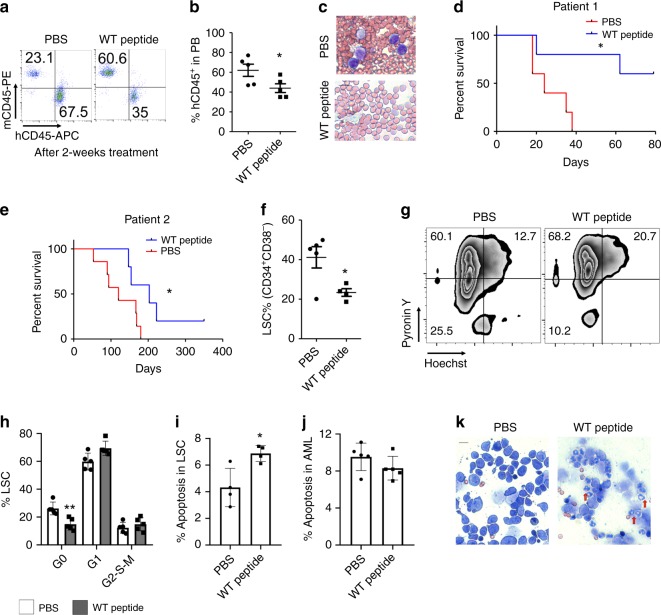
Inhibition of FOXM1 suppressed primary LSCs from MA9 patients. **a, b** Flow cytometry analyzing human AML cells ratio in PB cells from patient-derived xenograft (PDX) mice. Gating strategy is shown in (**a**) and summary data are presented in (**b**). **c** Wright-Giemsa-stained PB cells from PDX mice are shown, bar = 20 μM. **d, e** Kaplan–Meier survival curve of PDX mice for two patient samples, *n* = 5 mice for each group in (**d**), *n* = 5 mice for PBS or *n* = 7 mice for WT peptide treatment group. **f** Flow cytometric analysis of LSC ratio (CD34^+^CD38^−^) in PDX mice (*n* = 5). **g, h** Flow cytometric analysis of cell cycle of LSC in PDX mice. Gating strategy is shown in (**g**). The cells were stained with DNA Dye Hoechst and RNA Dye PyroninY. Double negative population indicated the G0 phase/quiescence, *n* = 5 mice for each group. **i** Flow cytometric analysis of apoptosis rate of LSCs in PDX mice, *n* = 4 mice for each group. **j** Flow cytometric analysis of apoptosis rate of total leukemia cells in PDX mice, *n* = 5 mice for each group. **k** Wright-Giemsa staining of human AML cells collected from PDX mice. Differentiated cells indicated by red arrow were present in WT peptide treatment mice. *p < 0.05, **p < 0.01, ***p < 0.001, bar = 20 μM, mean ± s.d., *t*-test or Log-rank (Mantel–Cox) Test for survival curve. The PDX mice were reconstituted with leukemia cells from primary MLL-r AML patients. All PDX mice were treated with WT peptide or vehicle PBS. Source data are provided as a Source Data file.

We next examined the effects of Foxm1 inhibition on engraftment of MA9.3 cells and primary LSCs from MLL-r AML patients in NSGS mice. Foxm1 inhibition by thiostrepton or Foxm1 knockdown by FOXM1-specific shRNAs inhibited the growth of MA9.3 cells in vivo (Fig. 8e, f), and significantly prolonged the survival of xenograft mice transplanted with MA9.3 cells (Fig. 8g, h). Moreover, we generated a cohort of PDX mice with primary leukemia cells from two MLL-r AML patients. When the engraftment of primary leukemia cells in vivo reached at approximately 5% in PB in xenografted mice, the mice were treated with FOXM1-specific peptide or PBS for 14 days. Of note, inhibition of FOXM1 suppressed growth of primary MA9 leukemia cells in vivo in mice, as evidenced by a lower percentage of human donor cells present in PB cells (Fig. 9a–c) in FOXM1-specific peptide-treated xenografted mice as compared to PBS-treated mice. The FOXM1-specific peptide-treated PDX mice had a significantly prolonged survival time (Fig. 9d, e) with a lower average of spleen weight (Supplementary Fig. 11e) as compared to PBS-treated control mice. More importantly, as determined by flow cytometric analysis of primary donor cells in BM in xenografted mice, we found that the percentage of CD34^+^CD38^−^ LSCs was markedly reduced in FOXM1-specific peptide-treated xenografted mice as compared to PBS-treated mice (Fig. 9f). In agreement with our findings in MA9-induced mouse model, inhibition of FOXM1 promoted LSCs to exit from quiescence and significantly induced apoptosis of LSCs but not mature leukemia cells (Fig. 9g–j). However, we also observed that FOXM1 inhibition induced the differentiation of primary human leukemia cells, as evidenced by presence of significant number of differentiated myeloid cells in BM from FOXM1-specific peptide-treated xenografted mice as compared to PBS-treated mice (Fig. 9k).

## Discussion

Our study has established a critical role of FOXM1 in regulation of survival, quiescence and self-renewal of LSCs in MLL-r AML. Foxm1 is a typical proliferation–associated transcription factor promoting S- and M-phase entry and controlling G1/S and G2/M transition^16,20,21,58–63^. Previous studies by other investigators suggest that *FOXM1* upregulation promotes tumorigenesis by potentiating tumor cell proliferation^22^. However, we found that Foxm1 loss promoted mouse and human MLL-r LSCs S phase entry and G0 phase exit in vitro and in vivo. By further in vivo functional studies, we demonstrated that Foxm1 loss reduced self-renewal of LSCs in vivo. Our results show that Foxm1 inhibits LSC proliferation, preserves LSC quiescence and promotes LSC self-renewal in MLL-r AML.

A number of genes including in BMI1, GFI1, TEL1, STAT5, and JUNB regulates self-renewal of both HSCs and LSCs^64^ whereas Pten^64^ and β-catenin^39,47,65^ have a distinct role in regulation of HSC and LSC self-renewal. Similar to its role in normal HSCs as we previously described^66^, Foxm1 regulates quiescence and self-renewal of LSCs, but through distinct molecular pathways, as evidenced by the finding that <2% of genes are commonly deregulated as a consequence of Foxm1 deletion in LSCs and HSCs. The expression of Nurr1, which is a key downstream mediator of Foxm1 in regulation of HSC quiescence and self-renewal^66^, is not changed in LSCs after induction of Foxm1 deletion. Our data showed that Foxm1 loss or inhibition leads to inactivation of the Wnt/β-catenin pathway by destabilizing β-catenin and promoting its degradation in mouse and human leukemia cells, which is distinct from the function of FOXM1 in promoting β-CATENIN nuclear localization instead of β-CATENIN expression in glioblastoma stem cell^24^. Previous data showed β-catenin is required for maintenance of MLL-LSC self-renewal and mediates chemoresistance of MLL-LSCs^39,47^. Our study shows a unique context-dependent role of FOXM1 in inhibition of β-catenin degradation in LSCs. We found that interaction between β-catenin and Foxm1 is critical for Foxm1–mediated β-catenin stabilization and the leukmeogneic activity of Foxm1, suggesting that β-catenin is a critical partner of Foxm1 that mediates function of Foxm1 in regulation of MLL LSC self-renewal, quiescence as well as chemoresistance. In addition, we found that Foxm1 is required for LSC but not HSC survival. Our finding that downregulation of genes including bcl2, and caspase 6, 7, which are involved in mitochondrial-mediated apoptosis, in LSCs but not HSCs, provide an explanation for the distinct role of Foxm1 in regulation of LSC survival.

The molecular mechanisms underlying the role of Foxm1 in chemoresistance remain unclear. We showed that Foxm1 loss and chemotherapeutic drugs had a synergistic effect on induction of apoptosis of mouse primary MA9-LSCs but not relatively mature leukemia cells. Of note, *Foxm1* loss significantly delayed the relapse of LSCs in MA9 leukemia mouse model. LSC quiescence provokes chemoresistance in AML^9,67^. Our study provides compelling evidence that Foxm1 contributes to the chemoresistance in AML by preserving LSC quiescence and enhancing LSC survival and self-renewal.

We demonstrated that FOXM1 inhibitors suppressed growth and induced apoptosis of human MLL-r leukemia cell lines but had much less effect or had a marginal effect on survival and growth of non-MLL-r leukemia cell lines such as K-562, Kasumi-3, HL-60, and U-937 when all cells were treated with FOXM1 inhibitors under the same condition. However, several other studies showed that inhibition of FOXM1 induced apoptosis of K-562, HL-60, and U-937 cells^27,51,68^. We found that increased doses of FOXM1 inhibitors also induced apoptosis of these non-MLL-r cell lines. FOXM1 is expressed at a relatively low level in AML patients with AML-ETO (Fig. 1a). We found that Foxm1 loss enhanced colony-forming ability of AML1–ETO9a-induced BM cells, raising the possibility that oncogenic activity of Foxm1 is dependent on cell context. Taken together, we conclude that MLL-r AML with a high FOXM1 expression is more sensitive to FOXM1 inhibition than other leukemia cell lines. However, inhibition of FOXM1 may have or not have an inhibitory effect on other non-MLL-r AMLs, indicating that personalized treatment of AML patients with FOXM1 inhibitors needs to be considered. Whether AMLs that carry other genetic mutations are sensitive to FOXM1 inhibition remained to be determined.

In conclusion, our in vitro and in vivo studies suggest that there is a therapeutic window by which that targeting FOXM1 by FOXM1 specific inhibitor eliminates MLL-r LSCs but has a minimal effect on normal HSCs. In addition, combination of traditional chemotherapy with FOXM1 inhibition likely improves the outcome of MLL-r AML patients and prevents or reduces the frequency of relapse after treatment, thereby benefiting MLL-r AML patients.

## Methods

### Mice

Foxm1^fl/fl^ mice^66^ were mated to Tie2-Cre or Mx1-Cre transgenic mice to generate Foxm1^fl/fl^ Tie2Cre and Foxm1^fl/fl^ Mx1Cre mice. Tie2-Cre expression is constant and Mx1-Cre expression can be induced by three times of 6 mg/kg poly(I:C) i.p. injection every other day. 6–8-week-old B6 mice and 8-week-old NSGS mice are recipient mice to receive mouse cells and human cells, respectively. Peripheral blood samples were collected from the tail vein and complete blood counts (CBC) were obtained with a Hemavet 950FS (Drew Scientific). All animals are free to access food and water. All animal research was approved by the University of Florida and University of Illinois at Chicago Institutional Animal Care and Use Committee.

### Cell culture

All leukemic cell lines were purchased from ATCC or DSMZ and were not authenticated by ourselves. All cell lines have been tested for mycoplasma contamination by vendors. Cell lines were expanded and cryogenically frozen upon acquisition to establish stocks in liquid nitrogen until use. The cell lines were cultured within 3–6 months after resuscitation. NOMO-1, MV-4-11, THP-1, and U-937 were cultured in RPMI-1640 (Corning) with 10% fetal bovine serum (FBS) (Sigma). Kasumi-1 and Kasumi-3 were cultured in RPMI-1640 with 20% FBS. K-562 cells were cultured in IMDM (Corning) with 10% FBS and HL-60 cells were cultured in IMDM (Corning) with 20% FBS. 293T cells were cultured in DMEM (VWR) with 10% FBS. UCSD-AML1 cells were cultured in RPMI-1640 with 20% FBS and 10 ng/ml GM-CSF. MA9.3 (human CD34^+^ cord blood cells transformed by MLL-AF9) and MA9.3RAS (human CD34^+^ with MLL-AF9 and mutant NRAS(G12D)) were established by Dr. James Mulloy^57^. MA9.3 cells were cultured in IMDM with 20% FBS and 10 ng/ml human SCF, TPO, FLT3L, IL3, and IL6, MA9.3RAS were cultured in the same medium without any cytokines. Mononuclear cells from healthy donors were isolated from umbilical cord blood (New York Blood Center) by using Ficoll-Paque Premium (GE healthcare), CD34^+^ cells were then purified from MNCs by human CD34 MicroBead Kit (Miltenyi Biotec). Primary CD34^+^ cells and all primary patient samples were cultured in IMDM with 20% FBS and human SCF, TPO, FLT3L, IL3, and IL6. Mouse BM cells (both MLL-AF9 transformed or not) were cultured in IMDM with 10% FBS and mouse SCF, IL3, and IL6. All cells were cultured at 37 °C with 5% CO_2_. Protocol for using patient samples for the described experiments was approved by University of Illinois Healthcare System Institutional Review Board policies and protocols (IRB#2010-0798:), cytogenetic information was provided in Supplementary Table 1.

### Plasmid construction

Primers of three human shRNA were annealed, followed by subcloning into pLKO-Tet-On inducible vectors at AgeI and EcoRI restriction enzyme sites. Luciferase was amplified from pGL4.20 (Promega) and cloned into MSCV-PIG by XhoI and EcoRI. Foxm1 was amplified and cloned into MSCV-puro at XhoI and EcoRI restriction enzyme sites. MSCV-YFP-MLL-AF9 was originally from Dr. Scott A. Armstrong (Harvard Medical School), and MSCV-Puro-GFP-AML1-ETO9a was originally from Dr. Dong-er Zhang (University of California San Diego).

### Virus preparation

For lentivirus production, hFOXM1-Tet-On shRNA together with package plasmids pMDG.2 and Δ8.91 (A gift from Dr Adrian J. Thrasher), were transfected into 293T cells by PEI. For retrovirus production, MSCV vector combined with PECO package vector, were transfected into 293T cells by PEI. The supernatant medium contained virus was collected at 48 and 72 h. The virus medium was filtered by 0.45 μM filter before use.

### Cell infection

Cells were mixed with virus medium, 4 μg/ml polybrene was added and cells were spinoculated for 3 h at 32 °C. The following day spinoculation was repeated. Then 1 μg/ml puromycin was added to select positive cells. For pLKO-Tet-On vector, 2 μg/ml doxycycline was added in the medium to induce shRNA express in vitro.

### Colony-forming assay

Mouse MLL-AF9 BM cells were mixed with Mouse Methylcellulose Base Media (R&D systems) with mouse 50 ng/ml SCF, 10 ng/ml IL3 and IL6. After vigorously vertexing, cells were plated into 24-well plate in triplicate. After 7 days, colony number was counted and serial replating was followed every other 7 days. For human cell lines and MA9.3RAS cells, Human Methylcellulose Base Media mixed with cells and the colony number was counted 7 days after plating. For human CD34^+^ cell, MA9.3 and primary patient samples, Human Methylcellulose Base Media contained human SCF, TPO, FLT3L, IL3, and IL6 were mixed with cells and colony number was counted after 10 days.

### Transplantation

For BM transplantation, 6–8-week Foxm1^fl/fl^ (WT) and Foxm1^fl/fl^TieCre or MxCre (KO) mice were injected 150 mg/kg 5-Fu (FRESENIUS KABI) by intraperitoneal injection. After 5 days, mice were sacrificed, and BM cells were collected. Twice spinoculation were performed to infect MLL-AF9. Infected cells were transplanted into 6–8 weeks sublethally irradiated B6 mice (6 Gary) by retro orbital injection. For serial transplantation, leukemic cells were collected from BM and transplanted into 6–8 weeks half-lethally irradiated B6 mice (6 Gary) by retro orbital injection. All human cells, both cell lines and primary patient samples, were transplanted into 8 weeks half-lethally irradiated NSGS mice (2.5 Gray) by tail vein injection. For pLKO-Tet-On shRNA cells transplantation in vivo, 2 mg/ml doxycycline and 10 mg/ml Sucrose were added in drinking water before transplantation and maintained through all lifetime.

### Drug treatment in vivo

FoxM1 inhibitor WT peptide (rrrrrrrrrKFVRSRRPRTASCALAFVN) and control Mut peptide (rrrrrrrrrSCALAFVN) were synthesized by Genemed Synthesis^69^. Peptides were dissolved in PBS and 10 mg/kg WT peptides were administrated by intraperitoneal injection for consecutive 14 days. Thisostrepton was purchased from Sigma and dissolved in DMSO. Before administration, PBS was added to dilute, and sonication was performed. 50 mg/kg thisostrepton were administered by intraperitoneal injection for consecutive 7 days^25^. The strategy of AraC (Zydus Hospira Oncology Private Ltd) and Doxorubicin (Teva Parenteral Medicines, Luc.) administration was mimic the human “7 + 3” regimen, in mice it is “5 + 3” regimen^44^. 100 mg/kg AraC was administered by intraperitoneal injection for consecutive 5 days and the last 3 days, 3 mg/kg Doxorubicin was administered by intraperitoneal injection.

### IVIS assay

Bioimaging of leukemia burden in vivo was performed by Xenogen IVIS Spectrum system (Caliper Life Science). Before performing, Luciferin (in vivo grade, VWR) was prepare in DPBS, and 150 mg/kg Luciferin was injected by i.p., after 10 min, luminescence signal can be detected for different time points. The signal data was analyzed by the Living Image (Caliper Life Science) software.

### Flow cytometric analysis

Suspended single cell was prepared from BM, spleen and peripheral blood. Red cells were lysed by ACK LYSING Buffer (VWR) before staining. Cells were incubated with antibodies in Facs buffer (2%FBS in PBS) on ice for 20 min at dark. All antibodies were listed in reporting summary. Anti- Gr-1, Ter119, B220, CD19, IgM, CD127, CD3e antibodies are lineage markers, streptavidin^−^PE-Cy5, anti- Sca1, c-Kit, CD34, and CD16, 32 antibodies are for leukemic stem cell L-GMP population analysis. Anti-c-Kit and Gr1 are for K^+^ G^−^ cKit^+^ Gr1^−^ population analysis. For cell quiescence (G0 population) staining, BM cells were incubated with 5 µg/ml Hoechst for 45 min at 37 °C, and then 1 µg/ml Pyronin Y was added to incubate at 37 °C for another 15 min. Furthermore, cells were stained with lineage cocktail, and then the cells were stained with LGMP or K^+^ G^−^ markers. For the detection of apoptosis, BM cells were stained with cell surface markers first, followed by Annexin V and DAPI staining. For BrdU cell cycle staining in vivo, 100 µl of 10 mg/ml BrdU was injected into mice by i.p. After 24 h, BM cells were collected and stained with antibodies against cell surface markers. The cells were then washed and stained with anti-BrdU antibodies and DAPI at RT for 20 min in dark. All cells were analyzed by Flow cytometry on CyAn bench-top analyzer (Beckman Coulter).

### Luciferase reporter assay

Mouse Foxm1 promoter (3.3 kb upstream of transcriptional start site) was cloned into PGL3.0 vector. Foxm1 promoter plasmids were co-transfected into 293 T cells by PEI (Polysciences, Inc.) with either MSCV empty vector or MSCV-MLL-AF9, as well as phRL-SV40 vector as an internal control. Cells were collected 48 h after transfection and both firefly and renilla luciferase activities were determined using Dual-Luciferase reporter assay system (Promega) on GLOMAX 20/20 LUMINOMETER (Promega).

### RNA-seq and RT-PCR analysis

BM cells from MLL-AF9-Foxm1^fl/fl^ and FoxM1-CKO mice were collected. After staining with antibodies against the cell surface markers, BM cells were sorted by MoFlo Astrios cell sorter (Beckman Coulter) according the following strategy: DAPI^−^YFP ^+ ^Lin^−^Sac1^−^cKit ^+^ CD34^ +^ CD16/32^+^. Total RNA was isolated by Trizol (invitrogen). RNA-seq was performed by UCLA Clinical Microarray Core (Los Angeles, CA). Library was prepared by Library Construction Kits (Clontech), and RNA-seq was performed on Illumina HiSeq 3000 system (Illumina, Inc.). RNA-seq data was analyzed, and the raw data was deposited in NCBI GEO. Gene-set enrichment analysis was performed with GSEA v2.0 software available from the broad institute (http://www.broad.mit.edu/gsea).

RT-PCR was performed on QS3 0.2 ML QPCR SYSTEM (Thermo Fisher), cDNA samples were prepared by Ovation® Pico WTA System V2 (NuGEN Technologies, Inc.). All primers were listed in Supplementary Table 2. All samples were run in triplicate. Amplification of Beta-Actin was used for sample normalization.

### Immunoprecipitation and Western Blotting

Cells were lysed in NETN Buffer (150 mM NaCl, 1 mM EDTA, 2 mM Tris-HCl and 0.5% NP-40, pH = 7.5) containing Protease Inhibitor Cocktail (Roche). After lysis on ice for 30 mins, the samples were spun, and supernatant were collected. FOXM1 (Santa Cruz, Cat#: SC-500) antibody plus Protein G Agarose beads (Sigma-Millipore) was added and samples were incubated on shaker at 4 °C for overnight. After washing with NETN Buffer, the samples were ready for western blot analysis. For ubiquitin detection, the cell pellets were lysed in 2% SDS PBS and incubated on 100 °C heat block for 10 min, 10 fold volume NETN buffer was added, followed by addition of β-CATENIN (BD) antibody and beads. Equal amount of protein was separated by 8% SDS-PAGE. Anti-FOXM1 primary antibody (Santa Cruz, Cat#: SC-500, 1:500 dilution), Anti- beta-Catenin (BD Biosciences, Cat#: 610153, 1:1000 dilution), anti - Ubiquitin antibody (Santa Cruz Biotechnology, Cat# sc-8017, 1:500 dilution), anti- Flag(Sigma-Aldrich, Cat# F3165, 1:5000 dilute) and anti-TUBULIN (Millipore, Cat#: 610153, 1:20000 dilution) were used for Western Blot analysis.

### Immunohistochemistry

MLL-AF9-Foxm1^fl/fl^ and MLL-AF9-Foxm1-CKO mice were sacrificed at the same time point. BM, liver, spleen and PB were collected. Cellular morphology of BM and PB smears were analyzed by May-Grünwald Giemsa staining. Liver and spleen section were stained with hematoxylin/eosin (H&E) by Northwestern University (Chicago, IL). All slides were evaluated in conventional light–field microscopy using an optical microscope (Olympus, Japan).

### Statistical analysis

Statistical significance was calculated using the two tailed Student’s *t* test. Results are expressed as the mean ± standard deviation (SD) for at least triplicate experiments. Measurements were taken from distinct samples. *P* values of < 0.05 were regarded as statistically significant which was calculated by Microsoft Excel or GraphPad Prism5. Kaplan–Meier survival curve was generated by GraphPad Prism5.

### Reporting summary

Further information on research design is available in the Nature Research Reporting Summary linked to this article.

## Supplementary information

Supplementary Information

Reporting Summary

## Data Availability

The RNA-seq data have been deposited in the NCBI SRA database under the accession code PRJNA515914 [https://www.ncbi.nlm.nih.gov/sra/?term=PRJNA515914]. The source data underlying Figs. 1d, 2a-b, 2e-f, 2h, 3b-h, 4a-h, 5a-d, 5f-g, 5i, 6c, 7a-h, 8a-h, 9b, 9d-f, 9h-j and Supplementary Figs. 1a, 2, 3, 4, 5a-d, 6a, 6c-g, 7b, 7e, 7g, 8b-c, 9a, 10a-c, 10e, 11a-e are provided as a Source Data file. Unprocessed gel images for Figs. 1c, 1e, 6f-j and Supplementary Figs. 7c-d, 7f, 8a, 9b, 10d are provided as a Source Data file. All the other data supporting the findings of this study are available within the article and its supplementary information files and from the corresponding author upon reasonable request. A reporting summary for this article is available as a Supplementary Information file.

## Source data

Source Data
