# Supplementary material for: FOXM1 regulates leukemia stem cell quiescence and survival in MLL-rearranged AML
Source: Nat Commun. 2020 Feb 17;11:928. doi: 10.1038/s41467-020-14590-9 (PMC7026046; doi:10.1038/s41467-020-14590-9)
Supplement: Supplementary file 1 — Supplementary Information [file 41467_2020_14590_MOESM1_ESM.pdf]

Supplementary Information

FOXM1 Regulates Leukemia Stem Cell Quiescence and Survival in MLL-Rearranged AML

Sheng et al.

Table of contents:

Supplementary figures 1-11

Supplementary tables 1-2

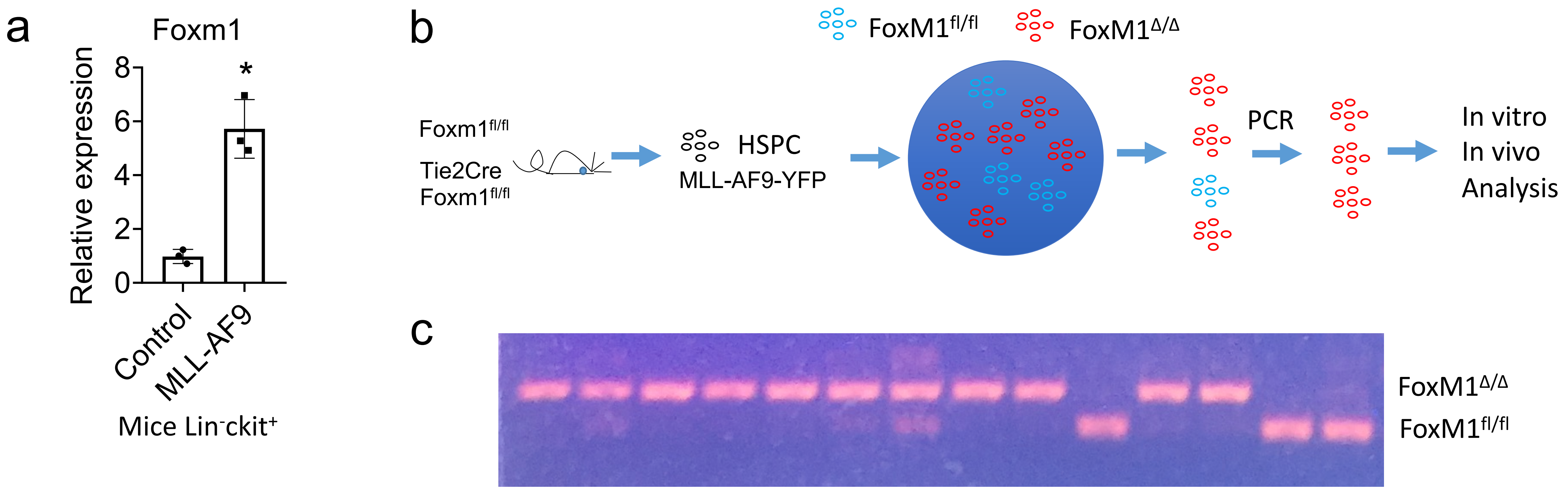

Supplementary Fig. 1 Foxm1 deletion was incomplete in Tie2Cre-Foxm1 mice.

(a) qPCR analysis of Foxm1 expression in mouse HSPCs, which were isolated from 6-8 weeks mice treated 5-Fu, infected with control plasmid or MLL-AF9-YFP. The average expression level of Foxm1 in control group was set as 1 for qPCR, n=3 mice for each group.
(b) Schematic diagram of selection of CFU-colonies with complete Foxm1 deletion.
(c) PCR analysis of Foxm1 deletion in single colonies from Methylcellulose medium.
*$p < 0.05$, mean ± s.d., t-test. Source data are provided as a Source Data file.

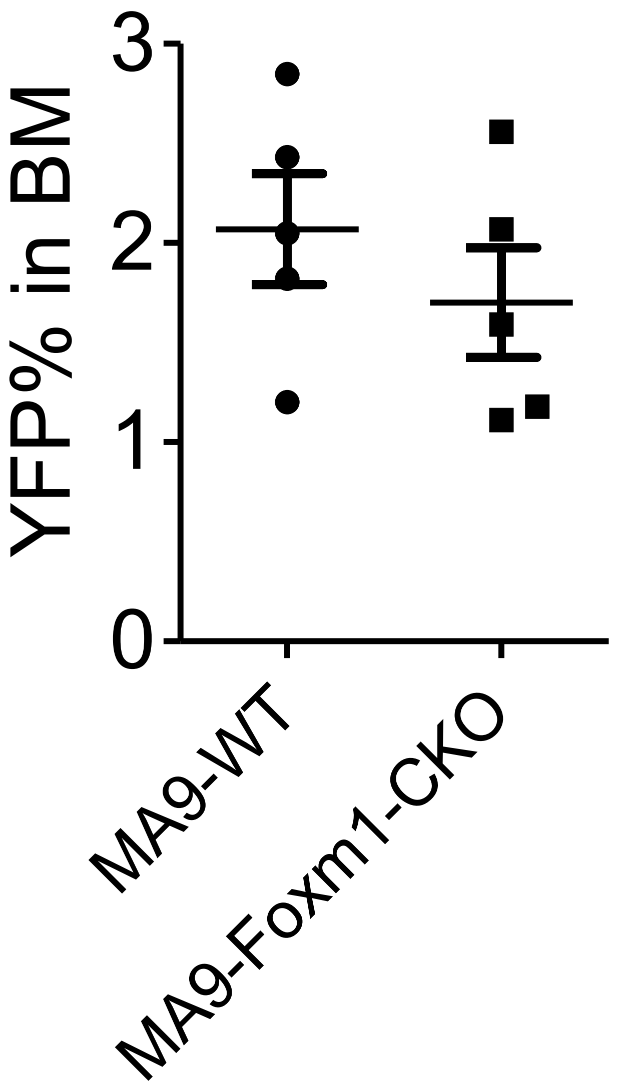

Supplementary Fig. 2 Homing ability of Leukemia cells.

MA9-Foxm1$^{fl/fl}$ or MA9-Foxm1-CKO cells were transplanted into lethally irradiated mice. The YFP+/leukemia cells in BM were determined by flow cytometric analysis 24 hours after transplantation, n=5 mice for each group, mean ± s.d., t-test. Source data are provided as a Source Data file.

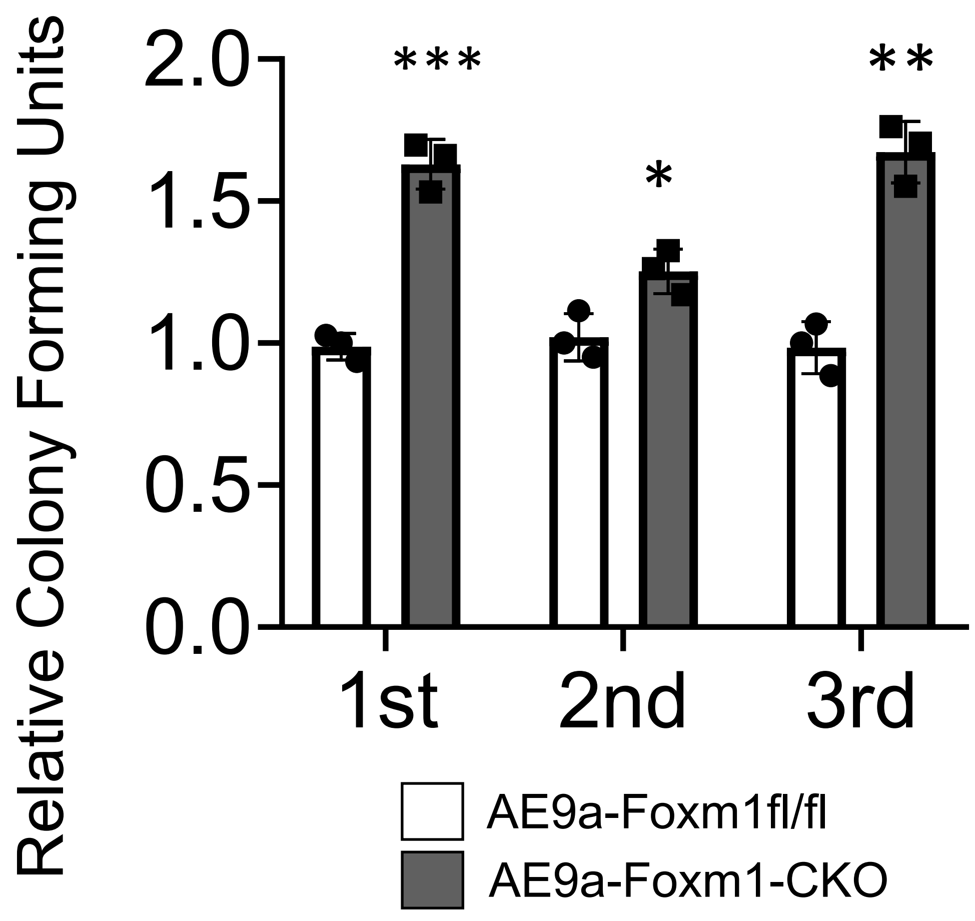

Supplementary Fig. 3 Colony forming ability of AML1-ETO9a-transformed hematopoietic stem/progenitor cells with or without Foxm1 KO.

The Lin^{-} BM cells isolated from Foxm1$^{fl/fl}$ and Foxm1-CKO BM cells mice were infected with retrovirus expressing MSCV-Puro-GFP-AML1-ETO9a. The infected cells were plated in MethoCult^{TM} medium containing cytokines in triplicate. The colonies were counted one week after plating, * $p<0.05$; **$p<0.01$; ***$p<0.001$, mean ± s.d., t-test. Source data are provided as a Source Data file.

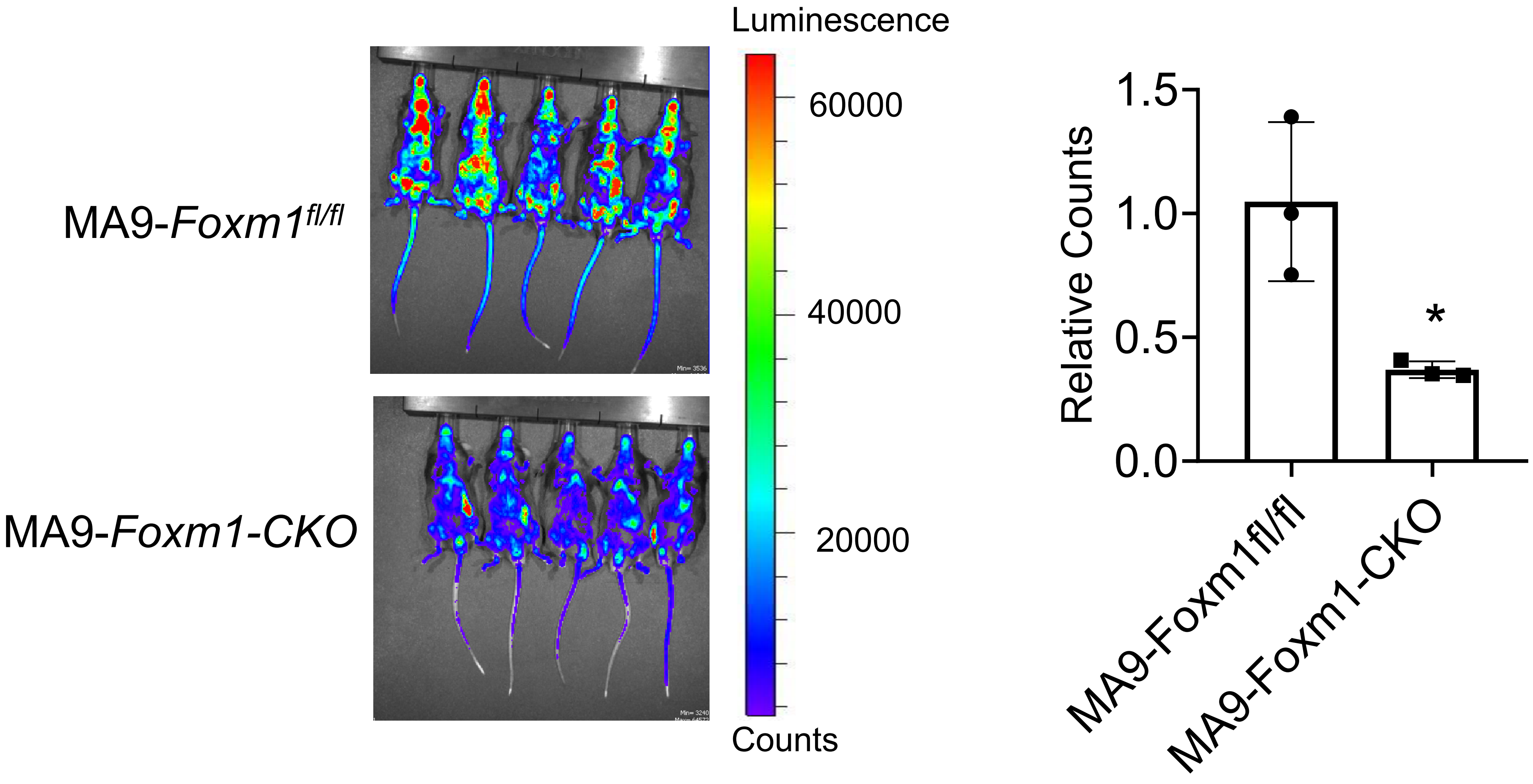

Supplementary Fig. 4 Leukemia burden of primary leukemia mice.

In vivo image system (IVIS) was used to determine the leukemia burden of primary MA9-Foxm1$^{fl/fl}$ or MA9-Foxm1-CKO recipient mice. BM cells were labeled with Luciferase and relative counts were shown. Mice were administrated substrate Luciferin before images were taken, n=3 mice for each group, *$p < 0.05$, mean ± s.d., t-test. Source data are provided as a Source Data file.

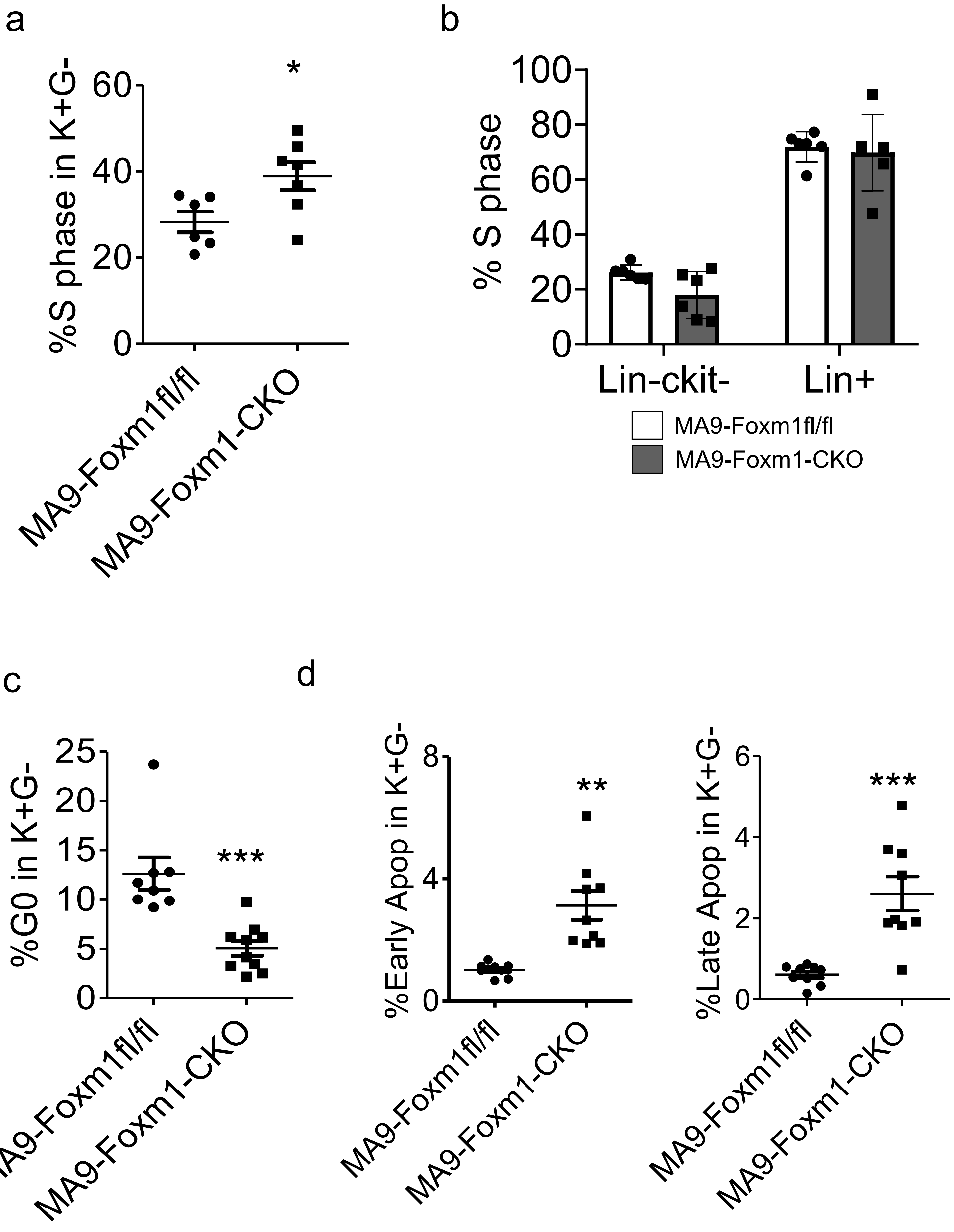

Supplementary Fig. 5 Foxm1 regulates quiescence and survival of c-Kit$^+$Gr1$^-$ cells.

(a) Flow cytometric analysis of cell cycle of c-Kit$^+$Gr1$^-$ cells in MA9-Foxm1$^{fl/fl}$ (n=6 mice) or MA9-Foxm1-CKO primary mice (n=7 mice).
(b) Flow cytometric analysis of cell cycle of Lin$^-$c-Kit$^-$ and Lin$^+$ cells in MA9-Foxm1$^{fl/fl}$ or MA9-Foxm1-CKO primary mice, n=6 mice for each group.
(c) Flow cytometric analysis of cell quiescence of c-Kit$^+$Gr1$^-$ cells in MA9-Foxm1$^{fl/fl}$ (n=8 mice) or MA9-Foxm1-CKO primary mice (n=10 mice).
(d) Flow cytometry analyzing the early and late apoptosis of c-Kit$^+$Gr1$^-$ cells in MA9-Foxm1$^{fl/fl}$ or MA9-Foxm1-CKO primary mice, n=9 mice for each group.
*p < 0.05, **p < 0.01, ***p < 0.001, mean ± s.d., t-test. Source data are provided as a Source Data file.

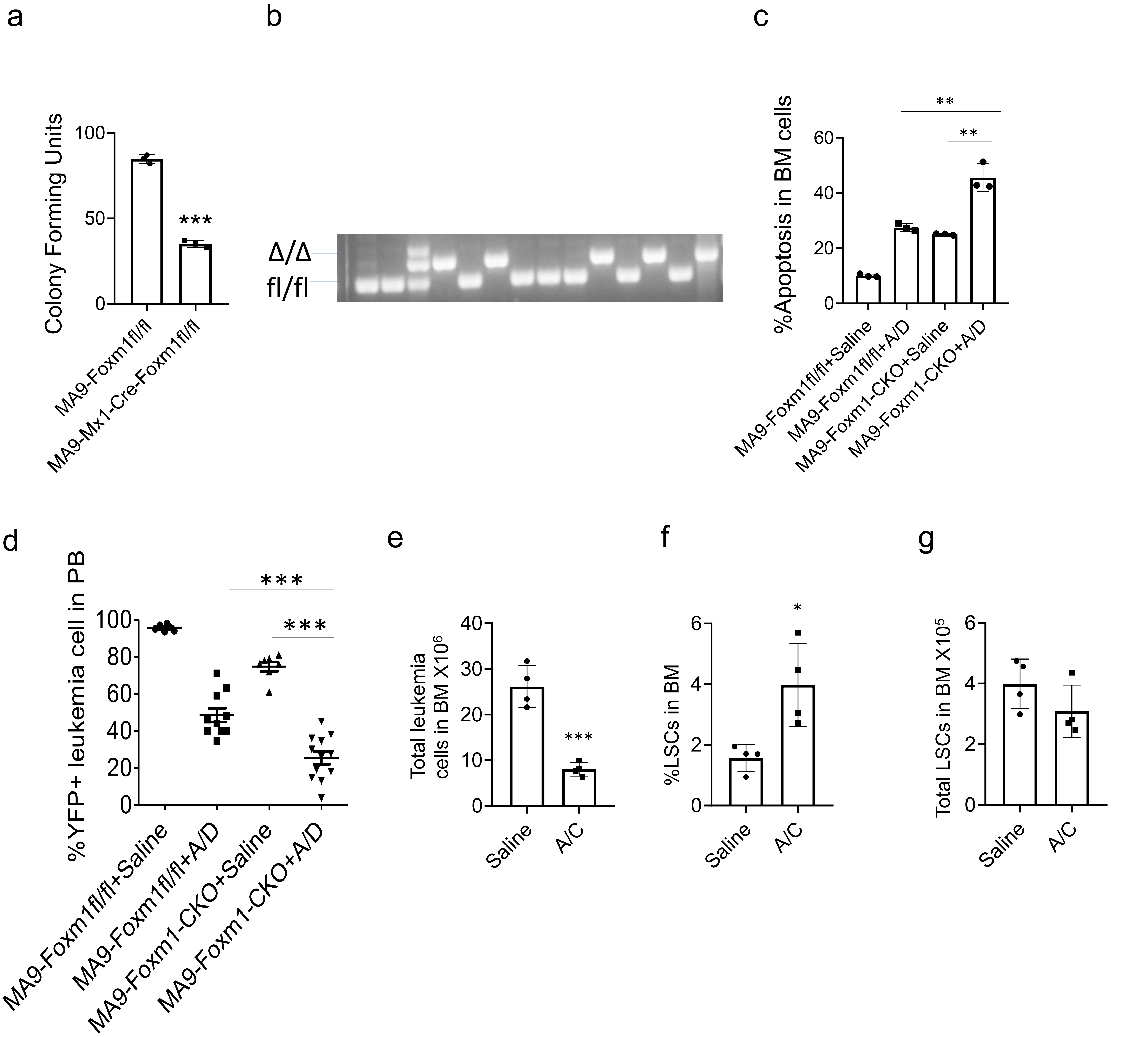

Supplementary Fig. 6 Foxm1 loss increases the sensitivity of MA9-transformed LSCs to chemotherapeutic drugs.

(a) Colony forming ability of MA9-Foxm1$^{fl/fl}$ or MA9-Mx1-CreFoxm1$^{f/fl}$ BM cells from the primary recipient mice, n=3 mice for each group.
(b) PCR analysis of Foxm1 deletion in single colonies from Methylcellulose medium.
(c) Flow cytometric analysis of apoptosis rate in MA9-Foxm1$^{fl/fl}$ or MA9-Foxm1-CKO BM cells from primary recipient mice treated with Saline or 0.5 μM Cytarabine/15nM Doxorubincin (Ara-C/Doxo), n=3 mice for each group.
(d) Flow cytometric analysis of leukemia cell (YFP+) ratio in PB collected from the recipient mice, which were reconstituted with MA9-Foxm1$^{fl/fl}$ or MA9-Foxm1-CKO BM cells and treated with Saline or “5+3” regimen, Ara-C and DOXO (A/D), n=6 mice for of MA9-Foxm1$^{fl/fl}$ +Saline group; n=10 mice for of MA9-Foxm1$^{fl/fl}$ +A/D group; n=7 mice for of MA9-Foxm1-CKO +Saline group, n=12 mice for of MA9-Foxm1-CKO +A/D group.
(e) Total leukemic cell (YFP+) number in BM at one day after treatment with Saline or AraC/Doxo, n=4 mice for each group.
(f) LSC frequency in BM at one day after treatment with Saline or AraC/Doxo, n=4 mice for each group.
(g) Total LSC number in BM at one day after treatment with Saline or AraC/Doxo, n=4 mice for each group, *p < 0.05, **p < 0.01, ***p < 0.001, mean ± s.d., t-test. Source data are provided as a Source Data file.

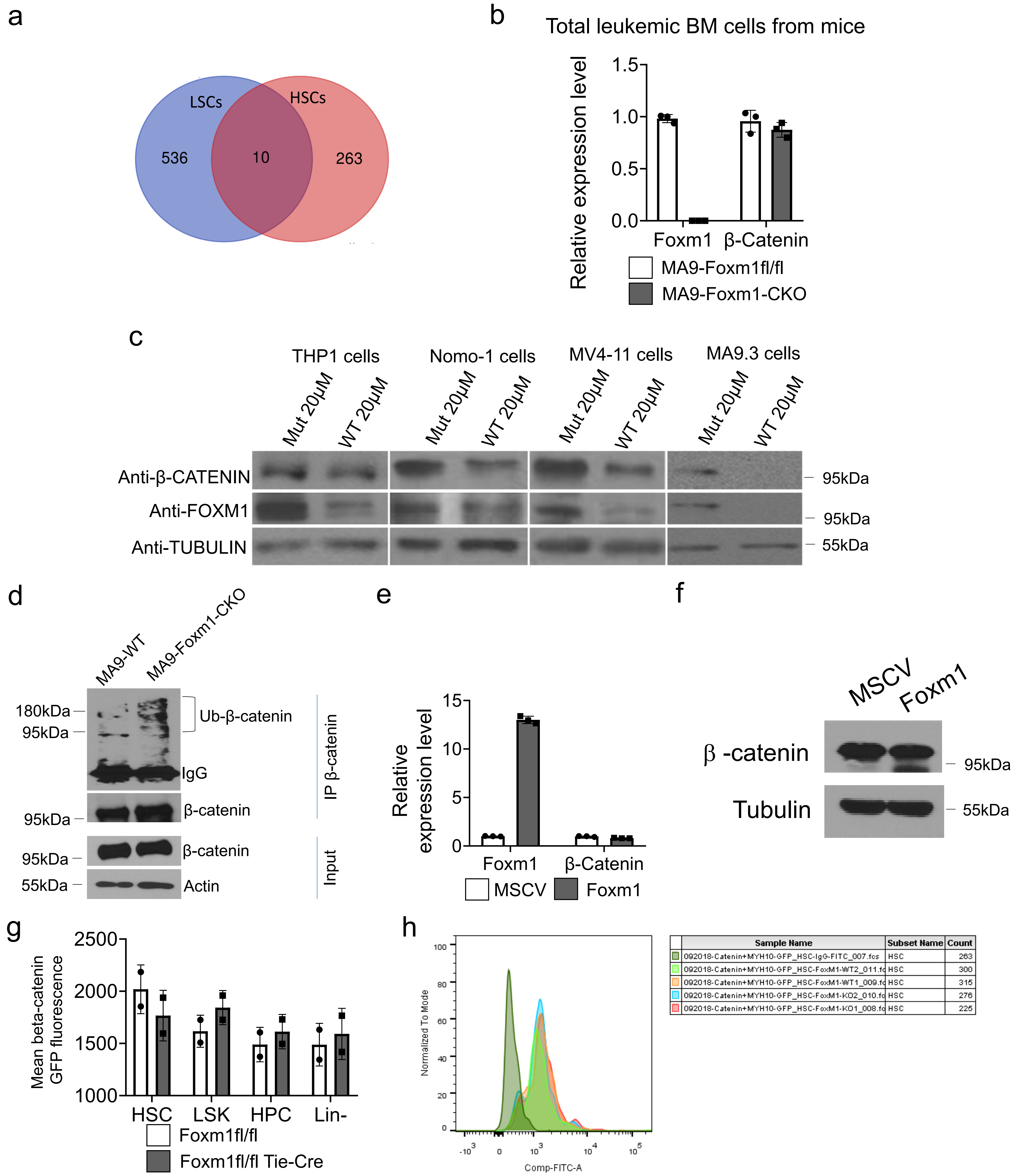

Supplementary Fig. 7 FOXM1 interacts β-Catenin and regulates β-catenin expression in MLL-rearranged leukemia cells.

(a) Venn diagram of differentially expressed genes in MA9-LSCs with or without presence of Foxm1 and HSCs with or without presence of Foxm1 gene.

(b)Relative expression of Foxm1 in total leukemic cells from MA9-Foxm1$^{fl/fl}$ and MA9-Foxm1-CKO, as determined by qRT-PCR. Results were normalized to those of Actb and are presented relative to those of Foxm1$^{fl/fl}$ control leukemic cells, n=3 mice for each group.

(c) Western Blot analysis of FOXM1 and β-CATENIN expression in human leukemia cells with expression of MLL fusion genes. The cells were treated with FOXM1-specific peptide or mutant peptide.

(d) The endogenous polyubiquitination of β-catenin was detected by anti-ubiquitin antibody in BM cells from MA9-Foxm1$^{fl/fl}$ and MA9-Foxm1-CKO. The cells were treated with 20μM MG-132 for six hours.

(e) Real-Time qPCR analysis of Foxm1 and β-Catenin expression level in mouse c-kit+ cells expressed MSCV vector or MSCV-Foxm1, n=3 mice for each group.

(f) WB was used to determine β-Catenin protein level in mouse c-kit+ cells expressed MSCV vector or MSCV-Foxm1.

(g-h) Intracellular flow cytometric analysis of β-Catenin protein level in Lin-, HPC, LSK and HSC population in Foxm1$^{fl/fl}$ or Foxm1$^{fl/fl}$ Tie2Cre mice, n=2 mice for each group. Summary data was shown in (g) and representing data was shown in (h).

Mean ± s.d., t-test. Source data are provided as a Source Data file.

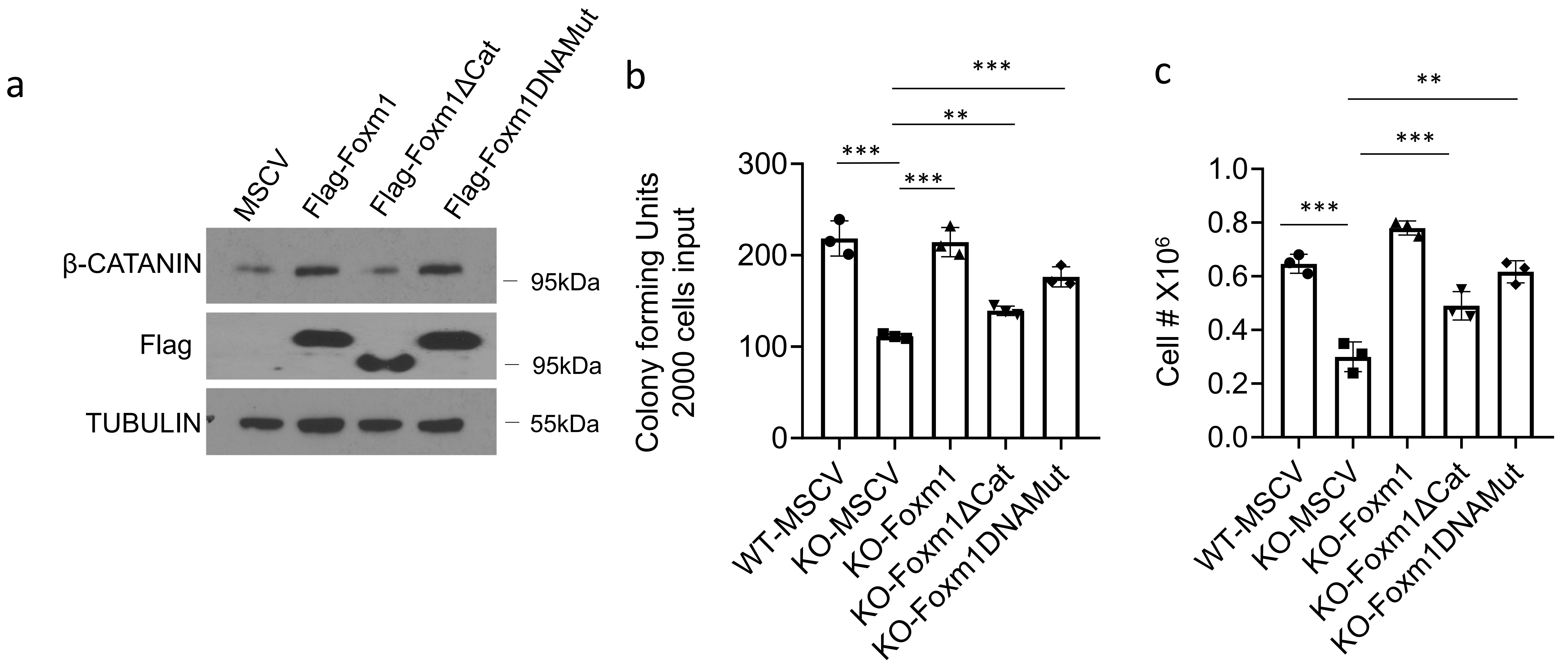

Supplementary Fig. 8 Leukemogenic function of Foxm1 is largely dependent on interaction between Foxm1 and β-Catenin.

(a) WB analysis of full length Foxm1, Foxm1 mutants as well as endogenous β-Catenin protein expression in MV4.11 cells expressing empty vector (MSCV), full length Foxm1 (Flag-Foxm1), Foxm1 deleted catenin interaction domain (Flag-Foxm1ΔCat) and Foxm1 DNA binding site mutant (Flag-Foxm1DNAMut) plasmids.

(b-c) Colony forming assay. MA9-WT cells infected with virus carrying MSCV empty vector and MA9-Foxm1-CKO cells infected with virus expressing empty vector (MSCV), full length Foxm1 (Flag-Foxm1), Flag-Foxm1ΔCat or Flag-Foxm1DNAMut vectors. were plated in plates containing methoCult^{TM} medium and cytokines. The colony forming units (b) and total cells (c) were determined on each plate one week after plating. **p<0.01; ***p < 0.001, mean ± s.d., t-test. Source data are provided as a Source Data file.

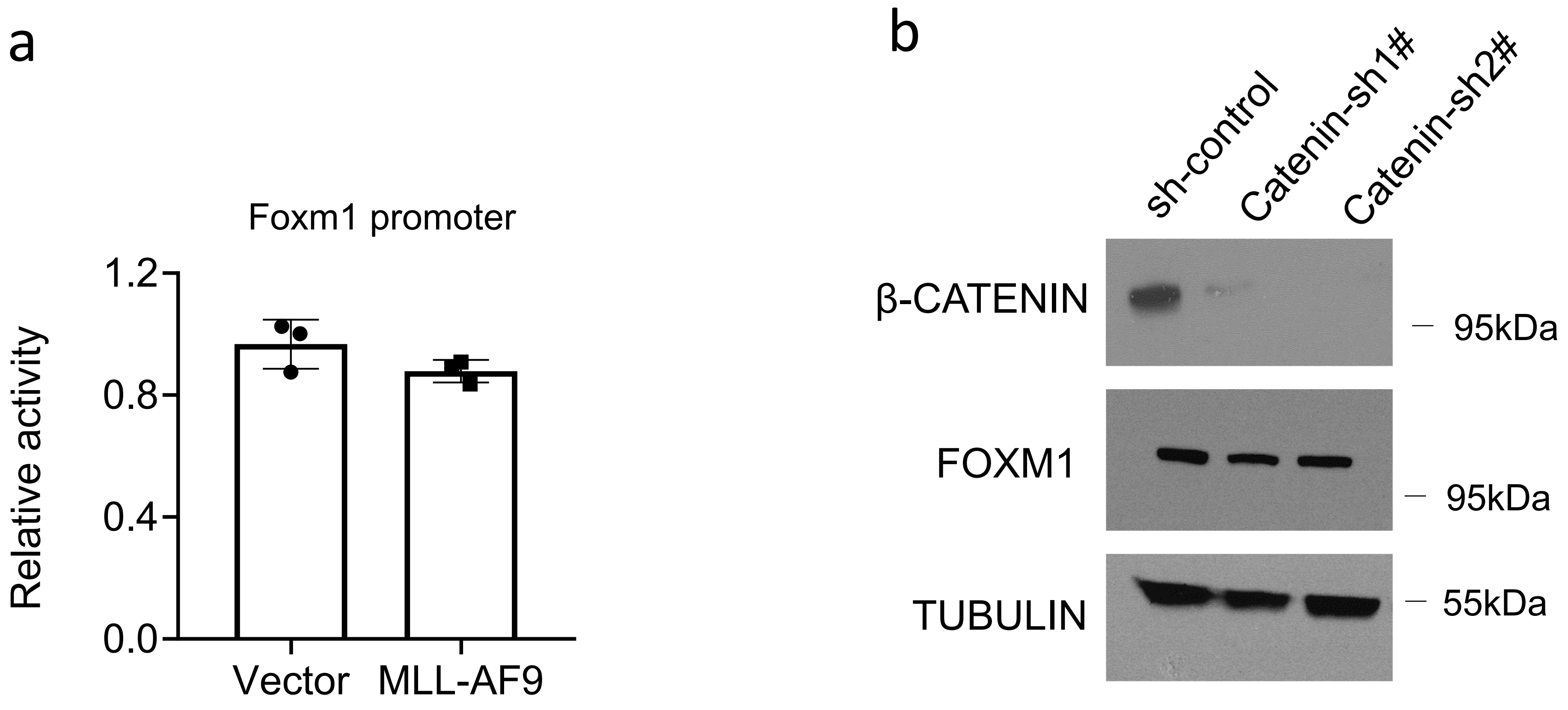

Supplementary Fig. 9 MLL-AF9 does not control Foxm1 expression directly or through β-CATENIN.

(a) Foxm1 promoter activity was evaluated in 293T cell by Dual-Luciferase reporter assay. Foxm1 promoter construct was co-transfected into 293T cells with either MSCV empty vector or MSCV-MLL-AF9, and phRL-SV40 vector as an internal control. After 48h, cells were lysed and promoter activity was determined by Dual-Luciferase assay.

(b) WB analysis of β-CATENIN and FOXM1 expression in MV4-11 expressing β-CATENIN-specific shRNAs or control vector. TUBULIN severed as an internal control , mean ± s.d., t-test. Source data are provided as a Source Data file.

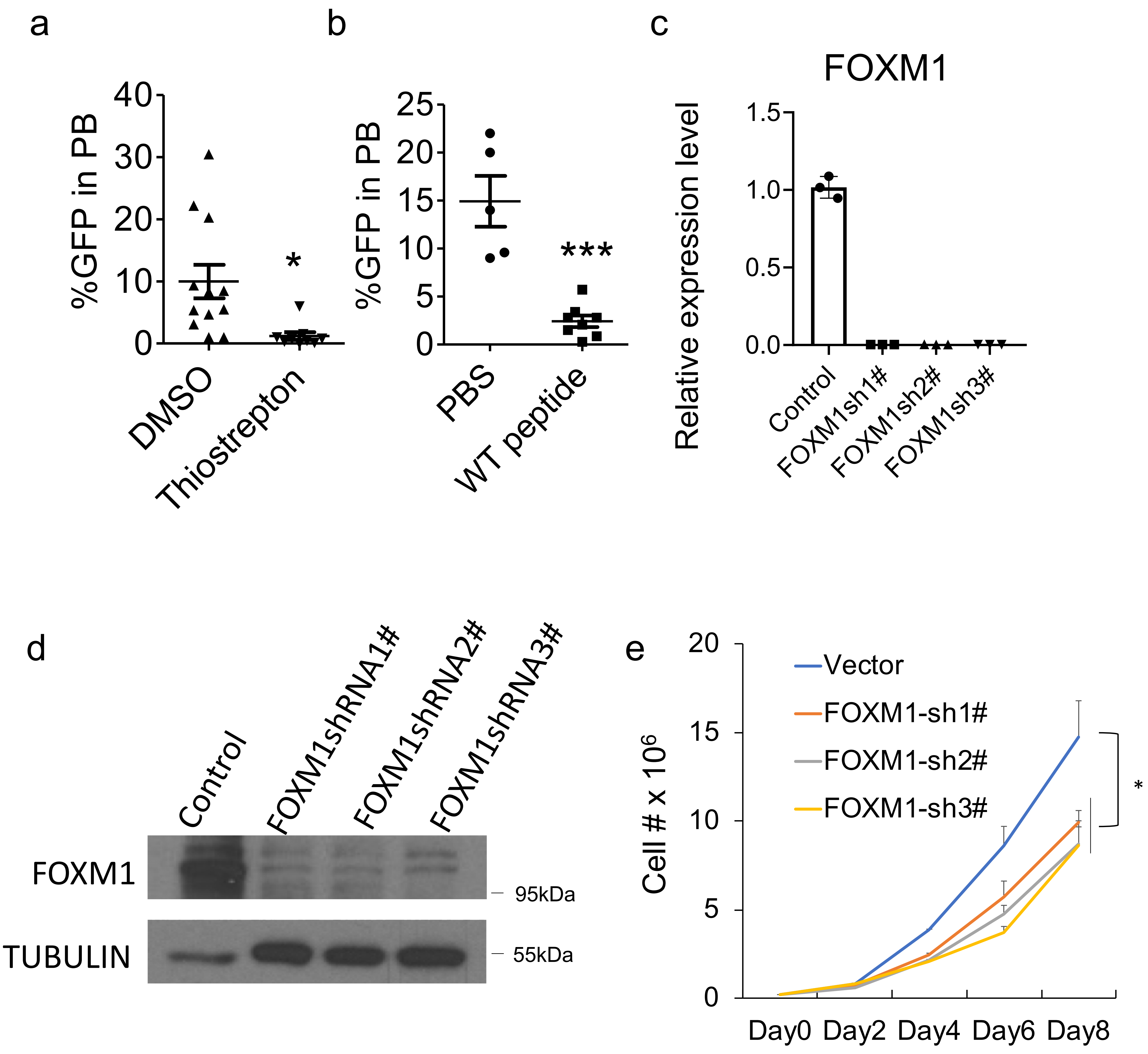

Supplementary Fig. 10 Inhibition of FOXM1 suppressed leukemogenic potential of MLL-rearranged leukemia cells in vitro and in vivo.

(a) Flow cytometric analysis of GFP percentage in PB cells collected from xenografted mice reconstituted with GFP labeled MV4-11 cells. The mice were treated with Thiostrepton (n=9 mice) or vehicle PBS (n=12 mice).
(b) Flow cytometric analysis of GFP percentage in PB cells collected from xenografted mice reconstituted with GFP labeled MV4-11 cells. The mice were treated with WT peptide (n=8 mice) or vehicle PBS (n=5 mice).
(c-d) Relative FOXM1 expression was determined by qRT-PCR (c) or West Blot analysis (d) in MV4-11 infected with inducible PLKO.1-Tet-on FOXM1-shRNA or control vector. 2 µg/ml doxycycline was added in the medium to induce shRNA expression for 2 days.
(e) Growth of MV4-11 cells infected with inducible PLKO.1-Tet-on FOXM1-shRNA or control vector. 2 µg/ml doxycycline was added in the medium to induce shRNA expression and cells were counted every two days. $^{*}p < 0.05$, $^{***}p < 0.001$, mean ± s.d., t-test. Source data are provided as a Source Data file.

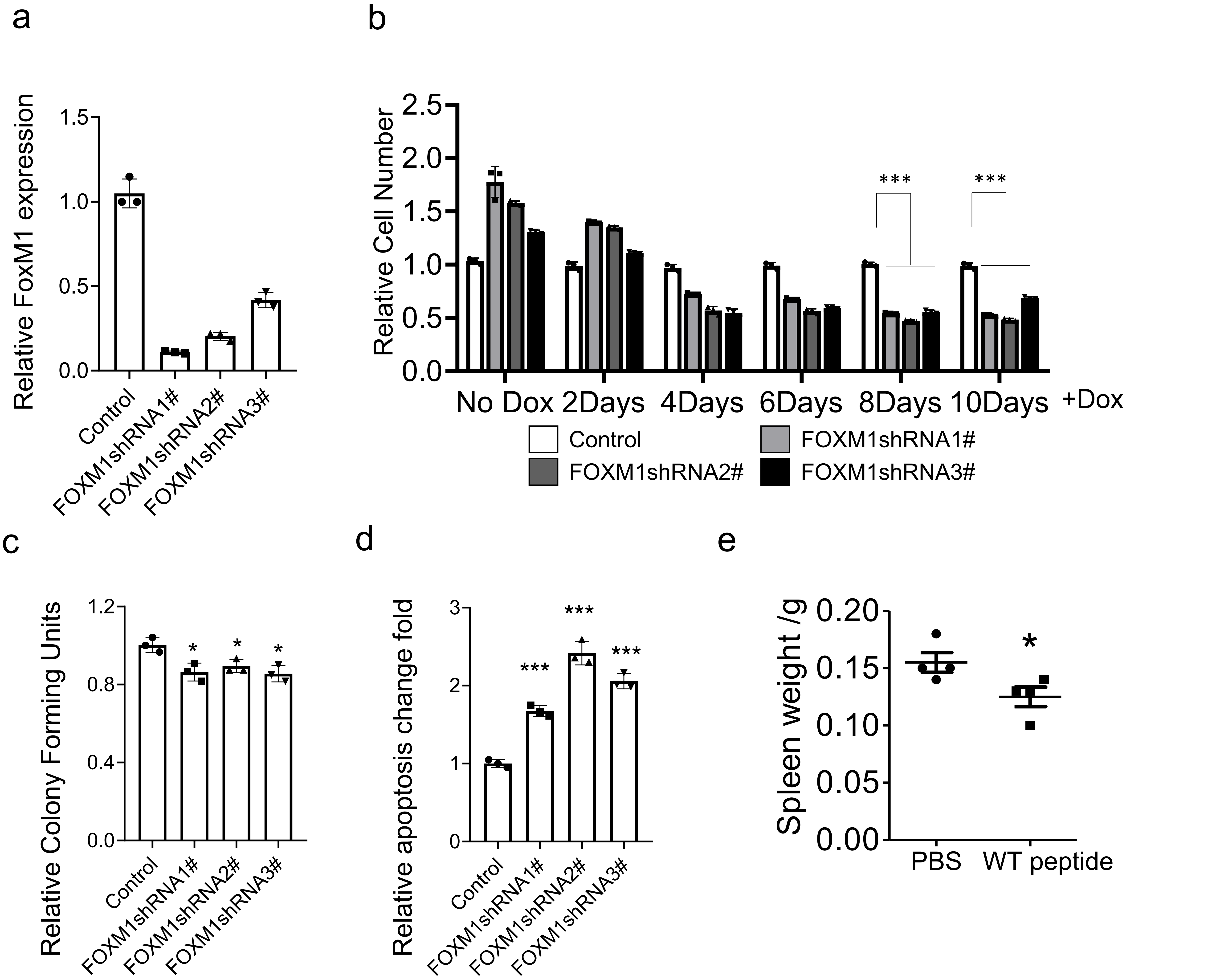

Supplementary Fig. 11 Inhibition of FOXM1 suppressed MA9-transformed CD34+ stem/progenitor cells and primary LSCs from patients in vitro and in vivo.

(a) Relative expression of FOXM1 was determined by qRT-PCR in MA9.3 cells infected with inducible PLKO.1-Tet-on FOXM1-shRNA or control vector. 2 μg/ml doxycycline was added in the medium to induce shRNA expression for 2 days.
(b) Growth rate of MA9.3 cells infected with inducible PLKO.1-Tet-on FOXM1-shRNA or control vector. 2 μg/ml doxycycline was added in the medium to induce shRNA expression and cells were counted every two days.
(c) Colony forming assay for MA9.3 cells infected with inducible PLKO.1-Tet-on FOXM1-shRNA or control vector. 2 μg/ml doxycycline was added in the methylcellulose medium to induce shRNA expression.
(d) Flow cytometric analysis of apoptotic MA9.3 cells infected with inducible PLKO.1-Tet-on FOXM1-shRNA or control vector. 2 μg/ml doxycycline was added in the medium to induce shRNA expression for 2 days.
(e) Spleen weight for PDX mice. The mice were treated with WT peptide or vehicle PBS, n=4 mice for each group.
*$p < 0.05$, ***$p < 0.001$, mean ± s.d., t-test. Source data are provided as a Source Data file.

Supplementary Table 1 Patient karyotype informarion

	Resource	Barcode	Cell Type	Sex	karyotype
Human: MLL-r AML 1#	Children's Oncology Group	#20727	BM	Male	46,XY,t(9;11)(p22;q23)[17]/46,XX[3]
Human: MLL-r AML 2#	Northwestern University	#17-0047	BM	Female	46,XX,t(9;11)(p22;q23)[20]
Human: MLL-r AML 3#	Northwestern University	#15-1756	BM	Female	51,XX,+6,+8,t(9;11)(p22;q23),+der(9)t(9;11)(p22;q23),+14,+19[18]

Supplementary Table 2 Primer list

Primers for subclone		
Luciferase-XhoI-F	CCGCTCGAGATGGATTACAAGGATGACGACGATAAGATGGAAGATGCCAAAAACAT	
Luciferase-EcoR1-R	CCGGAATTCTTACACGGCGATCTTGCCGC	construct luciferase in to PIG vector
hFoxM1-sh1-F	CCGGGCCAATCGTTCTCTGACAGAACTCGAGTTCTGTCAGAGAACGATTGGCTTTTTG	
hFoxM1-sh1-R	AATTCAAAAAGCCAATCGTTCTCTGACAGAACTCGAGTTCTGTCAGAGAACGATTGGC	construct human FOXM1 shRNA into PLKO.1-Tet on vector
hFoxM1-sh2-F	CCGGGCCCAACAGGAGTCTAATCAACTCGAGTTGATTAGACTCCTGTTGGGCTTTTTG	
hFoxM1-sh2-R	AATTCAAAAAGCCCAACAGGAGTCTAATCAACTCGAGTTGATTAGACTCCTGTTGGGC	construct human FOXM1 shRNA into PLKO.1-Tet on vector
hFoxM1-sh3-F	CCGGTTGCAGGGTGGTCCGTGTAAACTCGAGTTTACACGGACCACCCTGCAATTTTTG	
hFoxM1-sh3-R	AATTCAAAAATTGCAGGGTGGTCCGTGTAAACTCGAGTTTACACGGACCACCCTGCAA	construct human FOXM1 shRNA into PLKO.1-Tet on vector
Primers for real-time PCR		
m-RT-FoxM1-F	CACTTGGATTGAGGACCACTT	
m-RT-FoxM1-R	GTCGTTTCTGCTGTGATT	Detect mouse FoxM1 mRNA levels
m-RT-Nurr1-F	GTTTACCCTCGAAGCCGAAGAG	
m-RT-Nurr1-R	ATAGTCAGGGTTTGCCTGGAAC	Detect mouse Nurr1 mRNA levels
m-RT-P21-F	TGACCCACAGCAGAAGAG	
m-RT-P21-R	ACCAGCCTGACAGATTTCTA	Detect mouse P21 mRNA levels
m-RT-P27-F	TGGACCAAATGCCTGACTC	
m-RT-P27-R	GGGAACCGTCTGAAACATTTTC	Detect moues P27 mRNA levels
m-RT-Beta Actin-F	TGTGATGGTGGGAATGGGTCAG	
m-RT-Beta Actin-R	TTTGATGTCACGCACGATTTCC	Detect mouse Beta-actin mRNA levels
m-RT-Bcl2-F	TTGTGGCCTTCTTTGAGTTCGGTG	
m-RT-Bcl2-R	CTTCAGAGACAGCCAGGAGAAATC	Detect moues Bcl2 mRNA levels
m-RT-Caspase 6-F	CCAGACAGACAAGCTGGACA	
m-RT-Caspase 6-R	TGTACCAGGAGCCATTCACA	Detect moues Caspase 6 mRNA levels
m-RT-Caspase 7-F	CCGAGTGCCCACTTATCTGT	
m-RT-Caspase 7-R	ACCTGTCGCTTTGTCGAAGT	Detect moues Caspase 7 mRNA levels
m-RT-c-Myc-F	CTGGATTTCCTTTGGGCGTT	
m-RT-c-Myc-R	TGGTGAAGTTCACGTTGAGGG	Detect moues c-Myc mRNA levels
m-RT-beta-Catenin-F	CCGTTCGCCTTCATTATGGA	
m-RT-beta-Catenin-R	GGCAAGGTTTCGAATCAATCC	Detect moues beta-Catenin mRNA levels
h-RT-FOXM1-F	TTTAAGCACATTGCCAAGCCA	
h-RT-FOXM1-R	GGGGTGAATGGTCCAGAAGGA	Detect human FOXM1 mRNA levels
h-RT-Beta ACTIN-F	GCACAGAGCCTCGCCTT	
h-RT-Beta ACTIN-R	GTTGTCGACGACGAGCG	Detect human Beta ACTIN mRNA levels
Primers for Genotyping		
FoxM1 GT1	TGGCTTCCCAGCAGTACAAATC	TG1+TG2: Detect the FoxM1flox allele (280 bp) and FoxM1wildtype allele (226 bp)
FoxM1 GT2	TGCTTACAAAAGACACACTTGGACG	
FoxM1 GT3	TCTCGCTCAATTCCAAGACCAG	TG1+TG3: Detect deleted FoxM1 allele (400 bp)
Cre1	CTGCATTACCGGTCGATGCAAC	
Cre2	GCATTGCTGTCACTTGGTCGTG	Detect Cre allele (301bp), both Tie2 and Mx1 Cre
