# Supplementary material for: FOXM1 regulates leukemia stem cell quiescence and survival in MLL-rearranged AML
Source: Nat Commun. 2020 Feb 17;11:928. doi: 10.1038/s41467-020-14590-9 (PMC7026046; doi:10.1038/s41467-020-14590-9)
Supplement: Supplementary file 2 — Reporting Summary [file 41467_2020_14590_MOESM2_ESM.pdf]

nature research

Corresponding author(s): Zhijian Qian

Last updated by author(s): Dec 3, 2019

# Reporting Summary

Nature Research wishes to improve the reproducibility of the work that we publish. This form provides structure for consistency and transparency in reporting. For further information on Nature Research policies, see Authors & Referees and the Editorial Policy Checklist.

## Statistics

For all statistical analyses, confirm that the following items are present in the figure legend, table legend, main text, or Methods section.

| n/a | Confirmed | |
|---|---|---|
| ☐ | ☒ | The exact sample size (*n*) for each experimental group/condition, given as a discrete number and unit of measurement |
| ☐ | ☒ | A statement on whether measurements were taken from distinct samples or whether the same sample was measured repeatedly |
| ☐ | ☒ | The statistical test(s) used AND whether they are one- or two-sided<br>*Only common tests should be described solely by name; describe more complex techniques in the Methods section.* |
| ☒ | ☐ | A description of all covariates tested |
| ☒ | ☐ | A description of any assumptions or corrections, such as tests of normality and adjustment for multiple comparisons |
| ☐ | ☒ | A full description of the statistical parameters including central tendency (e.g. means) or other basic estimates (e.g. regression coefficient) AND variation (e.g. standard deviation) or associated estimates of uncertainty (e.g. confidence intervals) |
| ☒ | ☐ | For null hypothesis testing, the test statistic (e.g. *F*, *t*, *r*) with confidence intervals, effect sizes, degrees of freedom and *P* value noted<br>*Give P values as exact values whenever suitable.* |
| ☒ | ☐ | For Bayesian analysis, information on the choice of priors and Markov chain Monte Carlo settings |
| ☒ | ☐ | For hierarchical and complex designs, identification of the appropriate level for tests and full reporting of outcomes |
| ☒ | ☐ | Estimates of effect sizes (e.g. Cohen's *d*, Pearson's *r*), indicating how they were calculated |

*Our web collection on statistics for biologists contains articles on many of the points above.*

## Software and code

Policy information about availability of computer code

| | |
|---|---|
| Data collection | The IVIS signal data was collected by Living Image (Caliper Life Science) software. Flow cytometric data was collected by Summit Version 4.3. RNA-seq was collected from Illumina HiSeq 3000 system. |
| Data analysis | The IVIS signal data was analyzed by Living Image (Caliper Life Science) software. Flow cytometric data was analyzed by FlowJo Version 10. Gene set enrichment analysis was performed with GSEA v2.0 software. Statistical analyses were performed by Microsoft Excel or GraphPad Prism 5 |

For manuscripts utilizing custom algorithms or software that are central to the research but not yet described in published literature, software must be made available to editors/reviewers. We strongly encourage code deposition in a community repository (e.g. GitHub). See the Nature Research guidelines for submitting code & software for further information.

## Data

Policy information about availability of data

All manuscripts must include a data availability statement. This statement should provide the following information, where applicable:

- Accession codes, unique identifiers, or web links for publicly available datasets
- A list of figures that have associated raw data
- A description of any restrictions on data availability

The accession numbers for the RNA-Seq data is PRJNA515914 at NCBI SRA.

# Field-specific reporting

Please select the one below that is the best fit for your research. If you are not sure, read the appropriate sections before making your selection.

☒ Life sciences ☐ Behavioural & social sciences ☐ Ecological, evolutionary & environmental sciences

For a reference copy of the document with all sections, see nature.com/documents/nr-reporting-summary-flat.pdf

# Life sciences study design

All studies must disclose on these points even when the disclosure is negative.

| | |
|---|---|
| Sample size | The sample sizes were not predetermined by statistical method. For characterization of mice phenotypes, 3-7 mice were used for each group. For the other experiments for drug treatment, survival curve analysis, 3-15 mice were used for experiments. The specific number of mice used for each experiments were indicated in the figure legend. |
| Data exclusions | The recipient mice which failed to reconstitute hematopoietic system and died within 12 days after lethally-irradiated were excluded for survival analysis in the experiments in which recipient mice will be used. |
| Replication | All the experiments were performed in triplicate for each individual sample, and the results were reproduced in 3-5 independent experiments. |
| Randomization | The xenograft or recipient mice were randomly assigned to the experiment groups for treatment or without treatment of FOXM1 inhibitors or other drugs. Mice sex and age were kept controlled for each experiment. |
| Blinding | Investigator were not blinded to group allocation during data collection and analysis. |

# Reporting for specific materials, systems and methods

We require information from authors about some types of materials, experimental systems and methods used in many studies. Here, indicate whether each material, system or method listed is relevant to your study. If you are not sure if a list item applies to your research, read the appropriate section before selecting a response.

## Materials & experimental systems

| n/a | Involved in the study | |
|---|---|---|
| ☐ | ☒ | Antibodies |
| ☐ | ☒ | Eukaryotic cell lines |
| ☒ | ☐ | Palaeontology |
| ☐ | ☒ | Animals and other organisms |
| ☒ | ☐ | Human research participants |
| ☒ | ☐ | Clinical data |

## Methods

| n/a | Involved in the study | |
|---|---|---|
| ☒ | ☐ | ChIP-seq |
| ☐ | ☒ | Flow cytometry |
| ☒ | ☐ | MRI-based neuroimaging |

## Antibodies

Antibodies used

beta-CATENIN BD Biosciences Cat#: 610153, RRID: AB_397554
beta-TUBULIN Millipore Cat# 05-661, RRID:AB_309885
Ub Antibody (P4D1) Santa Cruz Biotechnology Cat# sc-8017, RRID: AB_628423
FOXM1 Santa Cruz Biotechnology Cat#: SC-500, RRID: AB_631521
FLAG Sigma-Aldrich Cat# F3165, RRID: AB_259529
Streptavidin PE-Cy5 Thermo Fisher Scientific Cat# 15-4317-82, RRID: AB_10116415
Mouse CD117 (c-Kit) APC-eFluor® 780 Thermo Fisher Scientific Cat# 47-1171-82, RRID: AB_1272177
Mouse Ly-6A/E (Sca-1) Monoclonal Antibody (D7), PE Thermo Fisher Scientific Cat# 12-5981-83, RRID: AB_466087
Annexin V Apoptosis Detection Kit APC Thermo Fisher Scientific Cat# 88-8007-74, RRID: AB_2575166
Annexin V - Apoptosis stain antibody FITC BD Biosciences Cat# 556419, RRID: AB_2665412
BrdU Monoclonal Antibody (BU20A), APC Thermo Fisher Scientific Cat# 17-5071-42, RRID: AB_11040534
BrdU Monoclonal Antibody (BU20A), FITC Thermo Fisher Scientific Cat# 11-5071-42, RRID: AB_11042627
Mouse CD16/CD32 PE-Cy7 Thermo Fisher Scientific Cat# 25-0161-82, RRID: AB_469598
Mouse CD34 Monoclonal Antibody (RAM34), eFluor 660 Thermo Fisher Scientific Cat# 50-0341-82, RRID: AB_10596826
Mouse CD117 (c-Kit) PE-Cy7 Thermo Fisher Scientific Cat# 25-1171-82, RRID: AB_469644
Mouse Ly-6G (Gr-1) APC-eFluor 780 Thermo Fisher Scientific Cat# 47-5931-82, RRID: AB_1518804
Mouse CD11b APC Thermo Fisher Scientific Cat# 17-0112-82, RRID: AB_469343
Mouse Ly-6G (Gr-1), PE Thermo Fisher Scientific Cat# 12-5931-82, RRID:AB_466045
Mouse CD71, PE Thermo Fisher Scientific Cat# 12-0711-82, RRID:AB_465740
Mouse TER-119 APC Thermo Fisher Scientific Cat# 17-5921-82, RRID:AB_469473
Mouse CD4 PE Thermo Fisher Scientific Cat# 12-0041-82, RRID:AB_465506

Mouse CD8a APC Thermo Fisher Scientific Cat# 17-0081-81, RRID:AB_469334
Mouse CD45 PE Thermo Fisher Scientific Cat# 12-0451-82, RRID:AB_465668
Human CD45 APC Thermo Fisher Scientific Cat# 17-9459-42, RRID:AB_10718532
Human CD34 APC Thermo Fisher Scientific Cat# 17-0349-41, RRID:AB_2016604
Human CD38, PE Thermo Fisher Scientific Cat# 12-0389-41, RRID:AB_10668491
Mouse CD3e Biotin Thermo Fisher Scientific Cat# 13-0033-86, RRID:AB_842773
Mouse/Human CD45R (B220) Biotin Thermo Fisher Scientific Cat# 13-0452-86, RRID:AB_466451
Mouse Ly-6G (Gr-1) Biotin Thermo Fisher Scientific Cat# 13-5931-86, RRID:AB_466802
Mouse CD127 Biotin Thermo Fisher Scientific Cat# 13-1271-85, RRID:AB_466589
Mouse CD19 Biotin Thermo Fisher Scientific Cat# 13-0193-86, RRID:AB_657655
Mouse TER-119 Biotin Thermo Fisher Scientific Cat# 13-5921-85, RRID:AB_466798
Rat IgM Isotype Control Biotin Thermo Fisher Scientific Cat# 13-4341-81, RRID:AB_470086
Mouse CD34 eFluor 450 Thermo Fisher Scientific Cat# 48-0341-82, RRID:AB_2043837
Anti-mouse IgG, HRP-linked Antibody Cell Signaling Technology Cat# 7076, RRID:AB_330924
Anti-rabbit IgG, HRP-linked Antibody Cell Signaling Technology Cat# 7074, RRID:AB_2099233

Validation

All primary antibodies were validated on manufacturer's website, related references and in our manuscript.

## Eukaryotic cell lines

Policy information about cell lines

Cell line source(s)

MV4-11 ATCC CRL-9591
RRID:CVCL_0064
THP-1 ATCC TIB-202
RRID:CVCL_0006
NOMO-1 DSMZ ACC-542
RRID:CVCL_1609
K562 ATCC CCL-243
RRID:CVCL_0004
Kasumi-1 ATCC CRL-2724
RRID:CVCL_0589
Kasumi-3 ATCC CRL-2725
RRID:CVCL_0612
HL-60 ATCC CCL-240
RRID:CVCL_0002
U-937 ATCC CRL-1593.2
RRID:CVCL_0007
UCSD-AML1 DSMZ ACC 691
RRID:CVCL_1853
MA9.3 A gift from Drs. Benjamin Mizukawa and James C. Mulloy N/A
MA9.3RAS A gift from Drs. Benjamin Mizukawa and James C. Mulloy N/A
HEK293T ATCC CRL-3216
RRID:CVCL_0063

Authentication

All leukemic cell lines were purchased from ATCC or DSMZ and were not authenticated by ourselves. Cell lines were expanded and cryogenically frozen upon acquisition to establish stocks in liquid nitrogen until use. The cell lines were cultured within 3 to 6 months after resuscitation.

Mycoplasma contamination

All cell lines have been tested for mycoplasma contamination by vendors.

Commonly misidentified lines
(See ICLAC register)

None commonly misidentified cell lines were used.

## Animals and other organisms

Policy information about studies involving animals; ARRIVE guidelines recommended for reporting animal research

Laboratory animals

6-8 weeks B6 mice and 8 weeks NSGS mice were used in our experiments, sex and age were matched when analysis were performed.

Wild animals

The study did not involve wild animals.

Field-collected samples

No samples were collected from field.

Ethics oversight

All animal research was approved by the University of Florida and University of Illinois at Chicago Institutional Animal Care and Use Committee.

Note that full information on the approval of the study protocol must also be provided in the manuscript.

# Flow Cytometry

## Plots

Confirm that:

☒ The axis labels state the marker and fluorochrome used (e.g. CD4-FITC).

☒ The axis scales are clearly visible. Include numbers along axes only for bottom left plot of group (a 'group' is an analysis of identical markers).

☒ All plots are contour plots with outliers or pseudocolor plots.

☒ A numerical value for number of cells or percentage (with statistics) is provided.

## Methodology

| | |
|---|---|
| Sample preparation | Suspended single cell was prepared from bone marrow, spleen and peripheral blood. Red cells were lysed by ACK LYSING Buffer (VWR) before staining. Cells were incubated with antibodies in Facs buffer (2%FBS in PBS) on ice for 20 minutes at dark. All antibodies were listed in KEY RESOURCES TABLE. Anti- Gr-1, Ter119, B220, CD19, IgM, CD127, CD3e antibodies are lineage markers, streptavidin-PE-Cy5, anti- Sca1, c-Kit, CD34 and CD16,32 antibodies are for leukemic stem cell L-GMP population analysis. Anti-cKit and Gr1 are for “K+G-” cKit+ Gr1- population analysis. For cell quiescence (G0 population) staining, BM cells were incubated with 5 µg/ml Hoechst for 45 min at 37°C, and then 1 µg/ml Pyronin Y was added to incubate at 37 °C for another 15 min. Furthermore, cells were stained with lineage cocktail, and then the cells were stained with LGMP or K+G- markers. For the detection of apoptosis, BM cells were stained with cell surface markers first, Annexin V staining was followed in its specific binding buffer, and DAPI was added at last. For BrdU cell cycle staining in vivo, 100 µl of 10 mg/ml BrdU was injected by i.p. and after 24hours, mice were sacrificed, and BM cells were collected. After staining the cell surface markers, BM cells were fixed and permeabilized by Cytofix/Cytoperm buffer (BD Pharmingen), followed 1 hours DNase I digestion. Then cells were washed and stained with anti-BrdU antibodies at RT for 20 minutes in dark. After adding 1mg/ml DAPI, cells are ready to analyze. All cells were analyzed by Flow cytometry on CyAn bench-top analyzer (Beckman Coulter). |
| Instrument | All cells were analyzed by Flow cytometry on CyAn bench-top analyzer (Beckman Coulter), sorted by MoFlo Astrios cell sorter. |
| Software | Flow cytometric data was collected by Summit Version 4.3 and analyzed by FlowJo Version 10. |
| Cell population abundance | Live cell > 95%, determined by DAPI negative in total BM cells. In leukemia mice, frequency of Lin- was increased up to 20-30% , frequency of c-Kit+Sca1- in Lin- is about 5%-10%. The frequency of each population in mice was shown in the representative diagrams in the figure or supplementary data. |
| Gating strategy | Anti- Gr-1, Ter119, B220, CD19, IgM, CD127, CD3e antibodies are lineage markers, streptavidin-PE-Cy5, anti- Sca1, c-Kit, CD34 and CD16,32 antibodies are for leukemic stem cell L-GMP population analysis, gating strategy was Lin-c-Kit+Sca1-CD34+FcR-Ɣ+. Anti-cKit and Gr1 are for “K+G-” cKit+ Gr1- population analysis. For cell quiescence (G0 population) staining, BM cells were stained Hoechst and PyroninY, and then the cells were stained with LGMP or K+G- markers, PyroninY and Hoechst double negative is G0. For the detection of apoptosis, BM cells were stained with cell surface markers first, Annexin V staining was followed , and DAPI was added at last, Annexin V positive cells are apoptotic cells. For BrdU cell cycle staining in vivo, after staining the cell surface markers, BM cells were fixed and permeabilized by Cytofix/Cytoperm buffer (BD Pharmingen), followed 1 hours DNase I digestion. Then cells were washed and stained with anti-BrdU antibodies at RT for 20 minutes in dark. After adding 1mg/ml DAPI, cells are ready to analyze, BrdU positive population are S phase. All gating strategy are shown in figures. |

☒ Tick this box to confirm that a figure exemplifying the gating strategy is provided in the Supplementary Information.
